# Association of Aβ deposition and regional synaptic density in early Alzheimer’s disease: a PET imaging study with [^11^C]UCB-J

**DOI:** 10.1186/s13195-020-00742-y

**Published:** 2021-01-05

**Authors:** Ryan S. O’Dell, Adam P. Mecca, Ming-Kai Chen, Mika Naganawa, Takuya Toyonaga, Yihuan Lu, Tyler A. Godek, Joanna E. Harris, Hugh H. Bartlett, Emmie R. Banks, Victoria L. Kominek, Wenzhen Zhao, Nabeel B. Nabulsi, Jim Ropchan, Yunpeng Ye, Brent C. Vander Wyk, Yiyun Huang, Amy F. T. Arnsten, Richard E. Carson, Christopher H. van Dyck

**Affiliations:** 1 Alzheimer’s Disease Research Unit, Yale University School of Medicine, One Church Street, 8th Floor, New Haven, CT 06510 USA; 2 Department of Psychiatry, Yale University School of Medicine, 300 George Street, New Haven, CT 06510 USA; 3 Department of Radiology and Biomedical Imaging, Yale University School of Medicine, P.O. Box 208048, New Haven, CT 06520 USA; 4 Program on Aging, Yale University School of Medicine, P.O. Box 207900, New Haven, CT 06520 USA; 5 Department of Neuroscience, Yale University School of Medicine, P.O. Box 208001, New Haven, CT 06520 USA; 6 Department of Neurology, Yale University School of Medicine, P.O. Box 208018, New Haven, CT 06520 USA

**Keywords:** SV2A, Synaptic density, Aβ, Alzheimer’s disease, [^11^C]UCB-J PET, [^11^C] PiB PET

## Abstract

**Background:**

Attempts to associate amyloid-β (Aβ) pathogenesis with synaptic loss in Alzheimer’s disease (AD) have thus far been limited to small numbers of postmortem studies. Aβ plaque burden is not well-correlated with indices of clinical severity or neurodegeneration—at least in the dementia stage—as deposition of Aβ reaches a ceiling. In this study, we examined in vivo the association between fibrillar Aβ deposition and synaptic density in early AD using positron emission tomography (PET). We hypothesized that global Aβ deposition would be more strongly inversely associated with hippocampal synaptic density in participants with amnestic mild cognitive impairment (aMCI; a stage of continued Aβ accumulation) compared to those with dementia (a stage of relative Aβ plateau).

**Methods:**

We measured SV2A binding ([^11^C]UCB-J) and Aβ deposition ([^11^C]PiB) in 14 participants with aMCI due to AD and 24 participants with mild AD dementia. Distribution volume ratios (*DVR*) with a cerebellar reference region were calculated for both tracers to investigate the association between global Aβ deposition and SV2A binding in hippocampus. Exploratory analyses examined correlations between both global and regional Aβ deposition and SV2A binding across a broad range of brain regions using both ROI- and surface-based approaches.

**Results:**

We observed a significant inverse association between global Aβ deposition and hippocampal SV2A binding in participants with aMCI (*r* = − 0.55, *P* = 0.04), but not mild dementia (*r* = 0.05, *P* = 0.82; difference statistically significant by Fisher *z* = − 1.80, *P* = 0.04). Exploratory analyses across other ROIs and whole brain analyses demonstrated no broad or consistent associations between global Aβ deposition and regional SV2A binding in either diagnostic group. ROI-based analyses of the association between regional Aβ deposition and SV2A binding also revealed no consistent pattern but suggested a “paradoxical” positive association between local Aβ deposition and SV2A binding in the hippocampus.

**Conclusions:**

Our findings lend support to a model in which fibrillar Aβ is still accumulating in the early stages of clinical disease but approaching a relative plateau, a point at which Aβ may uncouple from neurodegenerative processes including synaptic loss. Future research should investigate the relationship between Aβ deposition and synaptic loss in larger cohorts beginning preclinically and followed longitudinally in conjunction with other biomarkers.

## Introduction

The concept that Alzheimer’s disease (AD) is initiated by the progressive accumulation of the amyloid-β peptide (Aβ) in brain regions important for cognition is currently the leading theory of causation but remains controversial [1]. A loss of synapses has long been recognized as perhaps the strongest neuropathological correlate of cognitive impairment in AD [1–3]. A refinement of the amyloid hypothesis is thus based on convergent evidence that Aβ oligomers, the most neurotoxic Aβ species, impair both synaptic function (e.g., long-term potentiation) and synaptic structure (e.g., dendritic spines) [4, 5]. Aβ plaques, themselves comprised of fibrillary Aβ, are thought to have a penumbra of soluble Aβ oligomers in which synaptic density is low, whereas synapse number normalizes at greater distances from the plaque core [6]. Attempts to associate Aβ pathogenesis with synaptic loss have thus far been limited to a small number of postmortem [7, 8] and transgenic mouse [6, 9–11] studies.

The ability to assess synaptic density in vivo would be of great utility for tracking AD progression and monitoring the efficacy of potential therapies. A novel molecular target is synaptic vesicle glycoprotein 2 (SV2), an essential presynaptic vesicle membrane protein whose isoform SV2A is ubiquitously expressed in virtually all synapses [12, 13]. To assess the spatiotemporal distribution of synaptic density in vivo, a PET tracer for SV2A known as [^11^C]UCB-J has previously been developed and advanced for human studies [14–16]. The specific utility of SV2A imaging as a synaptic marker relevant to AD was exemplified by our initial study using this novel SV2A PET tracer, in which we reported significant reductions in SV2A hippocampal binding in patients with amnestic mild cognitive impairment (aMCI) and mild AD dementia [17]. In our subsequent study of [^11^C]UCB-J in early AD in a larger cohort, we observed more extensive cortical and subcortical reductions in SV2A binding, most pronounced in the hippocampus and entorhinal cortex and more widespread than reductions in gray matter volume [18]. When the AD group was separated into aMCI and dementia subgroups, these patterns were observed at both stages of disease [18]. These results are consistent with the subset of postmortem studies that have examined the prodromal or mild stages of AD [19–24]. These studies have focused primarily on hippocampus as the site of the earliest and most profound synaptic loss [19–21], consistent with the early degeneration of entorhinal cortical cells projecting via the perforant path to the hippocampus [25, 26].

As a general principle, Aβ plaque burden is not well-correlated with indices of symptom duration and severity—at least in the dementia stage—as deposition of Aβ reaches a ceiling [27–29]—suggesting a dynamic balance between Aβ deposition and clearance [28, 29]. In the era of Aβ PET imaging, this ceiling has been better defined longitudinally, and brain Aβ load has been shown to approach a “plateau” [30]. Longitudinal studies have generally demonstrated continued Aβ accumulation through the prodromal stages of AD [31–33], with minimal change by the time of conversion to AD dementia [31, 34] or in the dementia stage [35]. Therefore, in the prodromal stage of AD, when Aβ plaques are still accumulating, we might expect them to be associated with indices of severity, including synaptic loss—particularly in those brain regions that show marked early synaptic loss, such as hippocampus.

In this study, we examined the association between fibrillar Aβ deposition with [^11^C] PiB PET and synaptic density with [^11^C]UCB-J in early AD. We hypothesized that global Aβ deposition would be more strongly inversely associated with hippocampal synaptic density in the aMCI stage than the mild dementia stage. Given some in vitro evidence for local associations between Aβ plaques and synaptic abnormalities [6, 8, 10, 11], we also conducted exploratory correlational analyses of both global and regional Aβ deposition with synaptic density in a broad range of cortical and subcortical regions.

## Methods

Detailed methods and statistical analyses are further described in the Supplementary Methods (Additional file 1).

### Study participants and design

Individuals aged 55–85 years were screened to ensure diagnostic eligibility. Participants with dementia met diagnostic criteria for probable dementia due to AD [36], had a Clinical Dementia Rating (CDR) score of 0.5–1.0 points, and a Mini-Mental Status Examination (MMSE) score ≤ 26 points. Participants with MCI met diagnostic criteria for amnestic MCI [37], had a CDR score of 0.5 points, and a MMSE score of 24–30 points. All participants with dementia and aMCI demonstrated impaired episodic memory, as evidenced by a Logical Memory II (LMII) score 1.5 standard deviations below an education-adjusted norm. Cognitively normal (CN) participants had a CDR score of 0, an MMSE score > 26, and a normal education-adjusted LMII score. The Rey Auditory Verbal Learning Test (RAVLT) was also administered to generate an episodic memory score, calculated by averaging the RAVLT and LMII *z*-scores. *APOE* genotyping was performed as in our previous study [38]. Participants with dementia and aMCI were required to be Aβ+ and all CN participants Aβ−, according to their [^11^C] PiB scans (Additional file 1). All participants provided written informed consent as approved by the Yale University Human Investigation Committee prior to participating in the study.

### Brain imaging

T1-weighted magnetic resonance imaging (MRI) was performed to define regions of interest (ROI) and to perform partial volume correction (PVC) using the Iterative Yang (IY) algorithm [39, 40]. PET scans were performed on the HRRT (207 slices, resolution < 3 mm full width half max [FWHM]), the highest resolution human PET scanner [41]. List-mode data were reconstructed using the MOLAR algorithm [42] with event-by-event motion correction based on an optical detector (Vicra, NDI Systems, Waterloo, Canada) [43]. Dynamic [^11^C] PiB scans were acquired for 90 min following administration of a bolus of up to 555 MBq of tracer [38], while dynamic [^11^C]UCB-J scans were acquired for 60 min after administration of a bolus of up to 740 MBq [16]. Software motion correction was applied to the dynamic PET images using a mutual-information algorithm (FSL-FLIRT) to perform frame-by-frame registration to a summed image (0–10 min). A summed motion-corrected PET image was registered to each participant’s MRI. Cortical reconstruction and volumetric segmentation was performed using FreeSurfer [version 6.0] [44]. Specific ROIs utilized for [^11^C] PiB and [^11^C]UCB-J analyses in this study included medial temporal (entorhinal, hippocampus, parahippocampal, amygdala), prefrontal, lateral temporal, posterior cingulate/precuneus, anterior cingulate, lateral parietal, lateral occipital, medial occipital, and pericentral ROIs, as previously described (Supplementary Tables 1 & 2 from [18]). Global Aβ deposition was determined for a composite of regions commonly affected by Aβ deposition in AD which included prefrontal, lateral temporal, posterior cingulate/precuneus, and lateral parietal ROIs.

#### Tracer kinetic modeling

For [^11^C] PiB image analysis, parametric images of *BP*_ND_ were generated using a simplified reference tissue model—2 step (SRTM2) from 0 to 90 min [45] with whole cerebellum as the reference region as previously described [18, 38]. These values were then directly converted to distribution volume ratios (*DVR*), in that *DVR* = *BP*_ND_ + 1. For [^11^C]UCB-J image analysis, parametric images of *BP*_ND_ were generated using a SRTM2 from 0 to 60 min [45] and a small ROI (2 mL) in the core of the centrum semiovale (CS) as the reference region [46]. SRTM2 requires a global clearance rate constant of the reference region (${k}_2^{\prime }$), which was previously computed as a population average of *k*_2_ of the CS obtained using the 1TC model (${k}_2^{\prime }$ = 0.027 min^− 1^) [18]. As previously described, values of *DVR* using a whole cerebellum reference region were then computed for each voxel as (*BP*_ND_ + 1)/(*BP*_ND_ [cerebellum] + 1) [18], which was used as the primary outcome measure for [^11^C]UCB-J. In a separate sample, conversion of *BP*_ND_ with a CS reference region to values of *DVR* with a cerebellar reference region was validated by comparison against regional values of [^11^C]UCB-J *DVR* calculated directly with a cerebellar reference region using the SRTM2 model from 0 to 60 min and *DVR* calculated from the 1-tissue compartment (1TC) model using metabolite-corrected arterial plasma curves and a cerebellar reference region (Additional file 1, Supplementary Figure 1). For whole-cortex surface-based correlations between synaptic density and Aβ deposition, parametric PET images were co-registered to native subject space, sampled onto the cortical surface, and spatially smoothed with a 10 mm FWHM Gaussian kernel prior to statistical analysis.

### Statistical analyses

Statistical analyses are described in detail in the Supplementary Methods (Additional file 1). Briefly, characteristics of the participant groups were compared using *χ*^2^ test for categorical variables and unpaired *t*-tests for continuous variables. Separate linear mixed models were used to compare [^11^C] PiB *DVR* and [^11^C]UCB-J *DVR* across multiple ROIs between CN, aMCI, and dementia groups, with post hoc tests including ANOVAs within each ROI followed by unpaired *t*-tests for between-group comparisons within an ROI. The Benjamini-Hochberg procedure was used to control the false discovery rate (FDR) for multiple comparisons (12 comparisons for ROIs, and 3 comparisons for diagnostic groups). For the primary analysis of the association between global Aβ deposition and hippocampal synaptic density in participants with aMCI and dementia, separate univariate regression analyses were performed for each diagnostic group with correlation coefficients (Pearson *r*) and associated two-tailed *P* values reported for each model. For sensitivity analyses, separate multiple linear regression models were fit that also included covariates of age and sex. Based on our primary hypothesis of stronger correlation in aMCI than dementia, Fisher *z*-transformation was used to assess for significant differences in correlation coefficients between the aMCI and dementia groups, with one-tailed *P* values reported. Exploratory analyses assessed the relationships between global Aβ deposition and regional synaptic density, as well as intra-regional Aβ deposition and synaptic density (both regional- and surface-based approaches) using Pearson *r* correlation and associated two-tailed *P* values. Correction for multiple comparisons was not performed for exploratory analyses. The contribution of partial volume effects on all aforementioned analyses was evaluated through application of PVC (Additional file 1).

## Results

### Participant characteristics

The study sample consisted of 57 participants—14 with aMCI due to AD, 24 with mild AD dementia, and 19 who were CN. This sample substantially overlapped that in our previous study [18] but included 4 additional participants with mild AD dementia. Diagnostic groups were well balanced for age (F (2,54) = 0.30, *P* = 0.74) and sex (*χ*^2^ = 2.55, *P* = 0.28), with the CN group demonstrating significantly more years of education than the dementia group (Table 1; CN: 17.7 years ±2.1, dementia: 15.8 years ±2.4, unpaired *t*-test, *P* = 0.02). The symptomatic groups had clinical characteristics typical of aMCI and mild dementia with significant deficits in cognition (MMSE; aMCI: 26.3 ± 2.9, dementia: 21.5 ± 3.0) and function (CDR sum of boxes; aMCI: 2.3 ± 1.0, dementia: 5.3 ± 1.5) in comparison to the CN participants (MMSE = 29.2 ± 1.1, CDR sum of boxes = 0.0 ± 0.0). Regarding *APOE* genotype, typical of such samples, 21.1% of CN, 64.3% of aMCI, and 70.8% dementia participants carried at least one copy of *APOE* ε4.

**Table 1:** Participant demographics and test results

	Cognitively normal	Mild cognitive impairment	Mild dementia	F/***χ***^**2**^	***P***
**Participants (*n*)**	19	14	24		
**Sex (M/F)**	9/10	9/5	9/15	2.55	0.28
**Age (years)**	71.5 (7.6)	71.6 (4.2)	69.9 (9.2)	0.30	0.74
**Education (years)**	17.7 (2.1)	17.3 (1.9)	15.8 (2.4)*	4.71	0.013
**CDR-global**	0 (0)	0.5 (0)***	0.88 (0.22)***^,†††^	194.90	< 0.0001
**CDR-SB**	0 (0)	2.32 (1.03)***	5.25 (1.52) ***^,†††^	119.72	< 0.0001
**GDS**	0.68 (0.82)	2.79 (2.15)*	1.50 (1.84)	6.40	0.003
**UPSIT**	34.05 (5.86)	22.64 (9.79)**	21.09 (6.95)***	17.35	< 0.0001
**MMSE**	29.21 (1.13)	26.29 (2.89)*	21.46 (2.98)***^,†††^	52.88	< 0.0001
**LMII**	13.58 (4.38)	3.57 (2.98)***	0.25 (0.44)***^,†^	113.55	< 0.0001
**RAVLT-delay**	11.05 (2.80)	2.14 (2.71)***	0.29 (0.69)***^,†^	143.61	< 0.0001
**Episodic memory (*z*-score)**	1.25 (0.52)	−0.35 (0.39)***	−0.78 (0.08)***^,†^	177.54	< 0.0001
***APOE*** **ɛ4 copy number (*n*)**				14.95	0.005
**2 copies**	0 (0%)	5 (35.7%)	5 (20.8%)		
**1 copy**	4 (21.1%)	4 (28.6%)	12 (50.0%)		
**0 copies**	15 (78.9%)	5 (35.7%)	7 (29.2%)		

Data for continuous variables are mean (SD). Data for *APOE* copy number are n (percent). *F* statistics and *P* values are from one-way ANOVA significance tests. *χ*^2^ statistics and *P* values for counts are from *χ*^2^ significance tests. Post hoc unpaired *t*-tests after one-way ANOVA for continuous variables were Bonferroni corrected for 3 diagnostic groups. * denotes significant group differences between either amnestic mild cognitive impairment and cognitively normal or mild dementia and cognitively normal. * *P* < 0.05, ** *P* < 0.001, *** *P* < 0.0001. † denotes significant group differences between mild dementia and amnestic mild cognitive impairment. † *P* < 0.05, †† *P* < 0.001, ††† *P* < 0.0001. Episodic Memory was calculated by averaging the *z*-scores of the RAVLT and LMII. *Abbreviations*: *CDR-global* clinical dementia rating global score, *CDR-SB* clinical dementia rating sub of boxes, *MMSE* Mini-Mental State Examination, *LMII* Logical Memory II score, *RAVLT* Rey Auditory Verbal Learning Test, *GDS* Geriatric Depression Scale, *UPSIT* University of Pennsylvania Smell Identification Test, *APOE* Apolipoprotein E

### Distribution of synaptic density and Aβ deposition in normal cognition, aMCI, and dementia

**Fig. 1:**
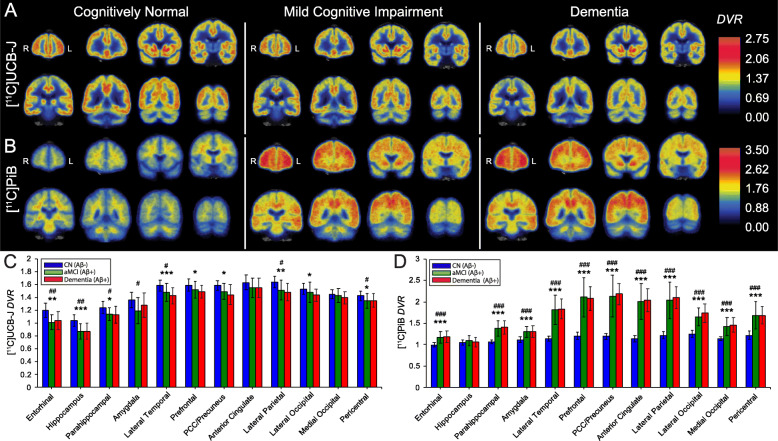
Comparison of SV2A and Aβ deposition in CN, aMCI, and dementia groups. Coronal sections of average parametric images of *DVR* for **a** [^11^C]UCB-J and **b** [^11^C] PiB in CN, aMCI, and dementia groups. Averaged images were created after co-registration to a common MNI template and overlaid on an MNI template T1 MRI. Parametric images adhere to radiological convention, with orientation denoted in the first coronal section of each image series. Quantification of between-group differences in **c** [^11^C]UCB-J *DVR* and **d** [^11^C] PiB *DVR* across all ROIs. One-way ANOVAs within each ROI with post hoc unpaired *t*-tests, FDR-corrected for multiple comparisons (3 comparisons for diagnostic groups), demonstrated significantly lower [^11^C]UCB-J *DVR* and significantly higher [^11^C] PiB *DVR* in both the aMCI and dementia participants (as compared to CN) across most analyzed regions. No group differences were observed in either SV2A or Aβ binding between aMCI and dementia participants across all analyzed ROIs. # denotes significant post hoc group differences between aMCI and CN. # *P* < 0.05, ## *P* < 0.001, ### *P* < 0.0001. * denotes significant group post hoc differences between dementia and CN. * *P* < 0.05, ** *P* < 0.001, *** *P* < 0.0001. Abbreviations: *DVR*, distribution volume ratio using a whole cerebellum reference region; PCC, posterior cingulate cortex, aMCI: amnestic mild cognitive impairment; CN: cognitively normal; MNI, Montreal Neurological Institute; MRI, magnetic resonance image; PET, positron emission tomography; ROI, region of interest; SV2A, synaptic vesicle glycoprotein 2A; PiB, Pittsburgh Compound B; FDR, false discovery rate

Analyses of both [^11^C]UCB-J and [^11^C] PiB *DVR* demonstrated significant effects of group ([^11^C]UCB-J: F (2,54) = 10.1, *P* = 0.0002, [^11^C]PiB: F (2,54) = 56.9, *P* < 0.0001), ROI ([^11^C]UCB-J: F (11,594) = 318.9, *P* < 0.0001, [^11^C]PiB: F (11,594) = 257.4, *P* < 0.0001), and group × ROI interaction ([^11^C]UCB-J: F (22,594) = 2.3, *P* = 0.001, [^11^C]PiB: F (22,594) = 43.3, *P* = < 0.0001) as predictors of SV2A and Aβ binding. Consistent with our recent publication [18], one-way ANOVA with post hoc, false discovery rate (FDR)-corrected unpaired *t*-tests revealed significant reductions of SV2A binding in both aMCI and dementia participants (compared to CN) across the majority of neocortical regions, with the exception of the anterior cingulate and medial occipital cortices (Fig. 1a, c, Supplementary Table 1). Group differences were largest in the hippocampus, entorhinal cortex, and lateral temporal cortex. The prefrontal, PCC/precuneus, and lateral occipital cortices also demonstrated significant reductions in SV2A binding in the dementia group, while non-significant trends of SV2A reduction were observed in aMCI participants (as compared to the CN group). One-way ANOVA with post hoc, FDR-corrected unpaired *t*-tests of [^11^C] PiB revealed significant and broadly distributed Aβ deposition across all neocortical regions in both aMCI and dementia (compared to CN) participants, with the exception of the hippocampus (Fig. 1b, d, Supplementary Table 2). No differences were observed in either SV2A or Aβ binding between aMCI and dementia groups across all analyzed ROIs. Average group images of *DVR* demonstrated visible reduction and deposition of SV2A and Aβ binding, respectively, in both aMCI and dementia groups (Fig. 1a, b), compared to the CN group.

Volumetric MRI was used to investigate gray matter volume differences between CN, MCI, and dementia groups (Supplementary Table 3). Correction for partial volume effects revealed continued significant reductions of SV2A binding in medial temporal regions in both participants with aMCI and dementia, as well as lateral parietotemporal reductions of SV2A binding in the dementia group only. After correction for partial volume effects, Aβ deposition remained significantly elevated across all neocortical regions in both aMCI and dementia participants, with the exception of the hippocampus (Supplementary Figure 2, Supplementary Tables 1 & 2).

### Association of global Aβ deposition and regional synaptic density in aMCI and dementia

**Fig. 2:**
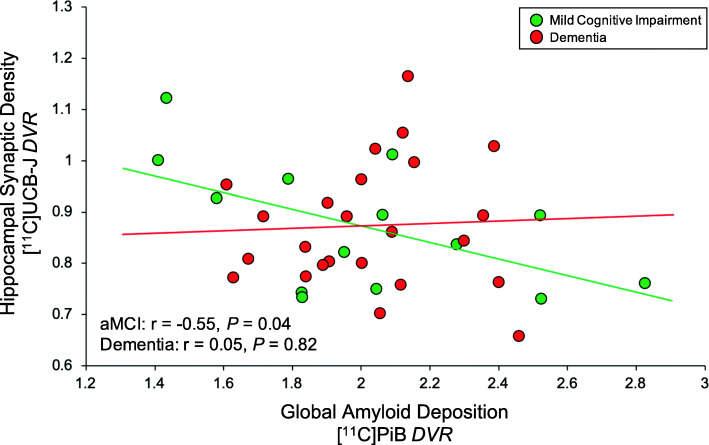
Correlation of global Aβ deposition and hippocampal SV2A in aMCI and dementia due to AD. Scatter plot with best-fit lines depicts a significant inverse association between global Aβ deposition and hippocampal SV2A in participants with aMCI (green) but not with dementia (red). Correlation coefficients were calculated from separate univariate linear regression analyses in each group with associated two-tailed *P* values, without correction for multiple comparisons. Global Aβ deposition was calculated by averaging values of [^11^C] PiB *DVR* from the bilateral prefrontal, lateral temporal, posterior cingulate/precuneus, and lateral parietal ROIs, weighted by volume. Green circles denote *DVR* values for aMCI participants, while red circles denote *DVR* values for participants with dementia. Abbreviations: *DVR*, distribution volume ratio using a whole cerebellum reference region; aMCI: amnestic mild cognitive impairment; SV2A, synaptic vesicle glycoprotein 2A; PiB, Pittsburgh Compound B

The primary analysis investigated the association of global Aβ deposition and hippocampal synaptic density (SV2A binding; Fig. 2). We hypothesized that in participants with aMCI (a stage of continued Aβ accumulation) but not in those with dementia (a stage of relative Aβ plateau), global Aβ deposition would be inversely associated with synaptic density in the hippocampus. Separate univariate linear regressions for aMCI and dementia groups demonstrated a significant inverse association in participants with aMCI (*r* = − 0.55, *P* = 0.04) but not in those with dementia (*r* = 0.05, *P* = 0.82). This difference between group correlation coefficients was significant (Fisher *z* = − 1.80, one-tailed *P* = 0.04). Addition of age and sex as covariates to this model reduced the previously observed significance in the aMCI group (R^2^ = 0.36, semi-partial correlation coefficient = − 0.48, *P* = 0.09), while the association between global Aβ deposition and hippocampal SV2A in the dementia group remained non-significant (R^2^ = 0.24, semi-partial correlation coefficient = 0.07, *P* = 0.72). When participants from the aMCI and dementia groups were pooled (*n* = 38), the inverse association between global Aβ and hippocampal SV2A was not significant (Pearson *r* = − 0.24, *P* = 0.58). Secondary exploratory analyses across multiple ROIs suggested no broad associations between global Aβ deposition and regional SV2A in either diagnostic group (Table 2). However, a nominal inverse association was observed between global Aβ deposition and lateral parietal SV2A in the dementia group (*r* = − 0.43, *P* = 0.03).

**Table 2:** Correlation of global Aβ deposition and regional SV2A in aMCI and dementia due to AD

	Mild cognitive impairment (***n*** = 14)		Dementia (***n*** = 24)		Fisher ***z***-transform	
**Primary region**	**Pearson** ***r***	***P***	**Pearson** ***r***	***P***	***z***	***P***
**Hippocampus**	−0.55	0.04*	0.05	0.82	−1.80	0.04
**Exploratory regions**	**Pearson** ***r***	***P***	**Pearson** ***r***	***P***	***z***	***P***
**Entorhinal**	−0.08	0.77	0.10	0.65	−0.49	0.31
**Parahippocampal**	−0.29	0.31	0.01	0.97	−0.83	0.20
**Amygdala**	−0.43	0.13	0.04	0.87	−1.34	0.09
**Lateral temporal**	−0.26	0.38	−0.10	0.65	−0.45	0.33
**Prefrontal**	−0.02	0.95	−0.21	0.32	0.52	0.30
**PCC/precuneus**	−0.12	0.69	−0.32	0.12	0.57	0.29
**Anterior cingulate**	0.07	0.80	−0.05	0.82	0.32	0.37
**Lateral parietal**	−0.18	0.53	−0.43	0.03*	0.75	0.23
**Lateral occipital**	−0.13	0.66	−0.32	0.13	0.54	0.30
**Medial occipital**	−0.10	0.73	−0.19	0.37	0.25	0.40
**Pericentral**	0.00	0.99	−0.17	0.44	0.46	0.32

Data are Pearson *r* and associated two-tailed *P* values obtained from separate univariate linear regression analyses in each group, uncorrected for multiple comparisons. Global Aβ deposition was calculated by averaging values of [^11^C] PiB *DVR* from the bilateral prefrontal, lateral temporal, posterior cingulate/precuneus, and lateral parietal ROIs, weighted by volume. Global Aβ deposition was then correlated with [^11^C]UCB-J *DVR* from the 4 medial temporal structures and 8 neocortical ROIs. To determine significant differences between group correlation coefficients, Fisher *z*-transformations and associated one-tailed *P* values were reported. * denotes significant correlation, with *P* < 0.05. *Abbreviations*: *PCC* posterior cingulate cortex, *PiB* Pittsburgh Compound B, *SV2A* synaptic vesicle glycoprotein 2A, *DVR* distribution volume ratio using a whole cerebellum reference region, *ROI* region of interest, *aMCI* amnestic mild cognitive impairment

**Fig. 3:**
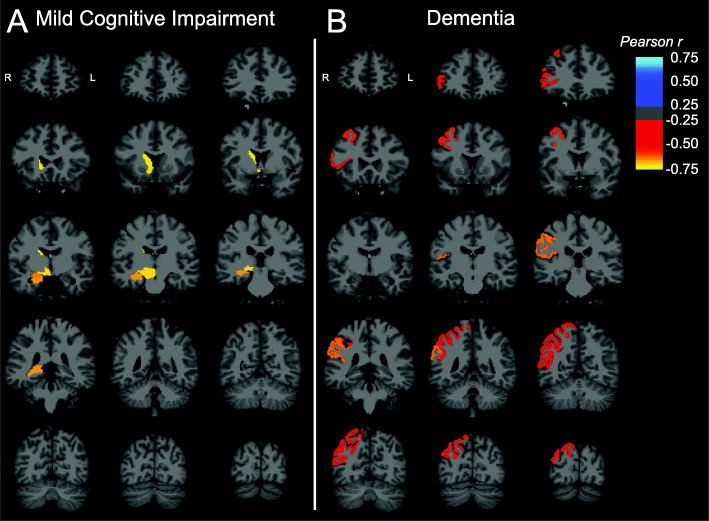
Brain maps of correlations between global Aβ deposition and SV2A in aMCI and dementia. Brain maps were created by producing images with the voxels in each FreeSurfer region set uniformly to the calculated Pearson *r* for that region and overlaid on an MNI template T1 MRI. Correlations were across all 84 lateralized FreeSurfer brain regions and displayed only for regions with uncorrected *P* < 0.05 in both the **a** aMCI and **b** dementia diagnostic groups. MR image slices adhere to radiological convention, with orientation denoted in the first coronal section of each image series. Abbreviations: *DVR*, distribution volume ratio using a whole cerebellum reference region; aMCI, amnestic mild cognitive impairment

Exploratory whole brain analyses were also performed on both a regional and surface-based level. On a regional level, the relationship between global Aβ deposition and SV2A binding in all FreeSurfer regions suggested negative correlations with right-sided subcortical structures in aMCI participants, including the hippocampus, amygdala, caudate, accumbens area, and ventral diencephalon (Fig. 3a). In participants with dementia, however, inverse associations were observed primarily with right-sided cortical regions, including caudal middle frontal, pars triangularis, supramarginal, superior parietal, and inferior parietal regions (Fig. 3b). Surface-based analyses of these same relationships revealed that global Aβ was negatively correlated with SV2A binding in a small cluster of vertices within the right lateral temporal cortex in participants with aMCI (Supplementary Figure 3A). In participants with dementia, by contrast, negative associations with SV2A binding were more widespread across the right frontal, right parietotemporal, and right lateral occipital cortices (Supplementary Figure 3B). No differences remained significant after permutation-based correction for multiple comparisons.

Correction for partial volume effects revealed similar results to those observed in non-corrected data across all correlational analyses, including associations between global Aβ deposition and hippocampal SV2A binding (Supplementary Figure 4), global Aβ deposition and SV2A binding in neocortical ROIs (Supplementary Table 4), and global Aβ deposition and regional SV2A binding in all FreeSurfer regions (Supplementary Figure 5).

### Association of regional Aβ deposition and regional synaptic density in aMCI and dementia

Additional exploratory analyses investigated regional Aβ deposition and suggested no broad associations with regional SV2A binding in either diagnostic group (Table 3). However, in the dementia group, a nominal inverse association was observed between medial occipital Aβ deposition and SV2A binding (*r* = − 0.46, *P* = 0.02). In addition, a positive correlation was detected between hippocampal Aβ deposition and synaptic density in the dementia group (*r* = 0.63, *P* = 0.001) and was also present if the aMCI and dementia groups were combined (*r* = 0.53, *P* = 0.001).

**Table 3:** Correlation of regional Aβ deposition and regional SV2A in aMCI and dementia due to AD

	Mild cognitive impairment (***n*** = 14)		Dementia (***n*** = 24)	
**Primary region**	**Pearson** ***r***	***P***	**Pearson** ***r***	***P***
**Hippocampus**	0.40	0.16	0.63	0.001*
**Exploratory regions**	**Pearson** ***r***	***P***	**Pearson** ***r***	***P***
**Entorhinal**	− 0.01	0.96	0.29	0.17
**Parahippocampal**	− 0.45	0.10	0.18	0.40
**Amygdala**	0.03	0.92	0.35	0.09
**Lateral temporal**	− 0.24	0.40	− 0.10	0.64
**Prefrontal**	− 0.04	0.88	− 0.28	0.19
**PCC/precuneus**	− 0.06	0.83	− 0.23	0.29
**Anterior cingulate**	0.14	0.63	0.02	0.93
**Lateral parietal**	− 0.13	0.65	− 0.26	0.22
**Lateral occipital**	− 0.02	0.96	− 0.32	0.12
**Medial occipital**	0.03	0.91	− 0.46	0.02*
**Pericentral**	0.09	0.77	− 0.12	0.58

Data are Pearson *r* and associated two-tailed *P* values obtained from separate univariate linear regression analyses in each group, uncorrected for multiple comparisons. Correlations between regional [^11^C] PiB and [^11^C]UCB-J *DVR* were assessed within 4 medial temporal structures and 8 neocortical ROIs. * denotes *P* < 0.05. *Abbreviations*: *PCC* posterior cingulate cortex, *PiB* Pittsburgh Compound B, *SV2A* synaptic vesicle glycoprotein 2A, *DVR* distribution volume ratio using a whole cerebellum reference region, *ROI* region of interest, *aMCI* amnestic mild cognitive impairment

**Fig. 4:**
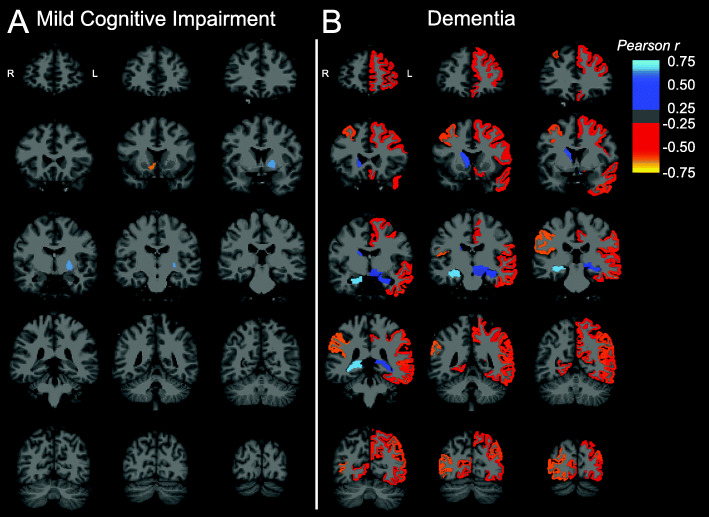
Brain maps of correlations between regional Aβ deposition and SV2A in aMCI and dementia. Brain maps were created by producing images with the voxels in each FreeSurfer region set uniformly to the calculated Pearson *r* for that region and overlaid on an MNI template T1 MRI. Correlations were across all 84 lateralized FreeSurfer brain regions and displayed only for regions with uncorrected *P* < 0.05 in both the **a** aMCI and **b** dementia diagnostic groups. MR image slices adhere to radiological convention, with orientation denoted in the first coronal section of each image series. Abbreviations: *DVR*, distribution volume ratio using a whole cerebellum reference region; aMCI, amnestic mild cognitive impairment

Analyses investigating the relationship between regional Aβ deposition and synaptic density in all FreeSurfer regions suggested few correlations in aMCI participants (Fig. 4a), while analyses restricted to participants with dementia revealed inverse associations across bilateral prefrontal, temporal, and parietal, and occipital (both medial and lateral) cortical regions (Fig. 4b). These inverse associations were strongest in right-hemisphere regions. In addition, consistent with the previous analysis, positive correlations were observed in the bilateral hippocampi of dementia participants.

Correction for partial volume effects yielded similar results to those observed in non-corrected data across all correlational analyses, including associations between regional Aβ deposition and SV2A binding in neocortical ROIs (Supplementary Table 5) and regional Aβ deposition and regional SV2A binding in all FreeSurfer regions (Supplementary Figure 6).

## Discussion

In this study, we investigated the association of cerebral Aβ deposition using [^11^C] PiB and regional synaptic density using [^11^C]UCB-J. Overall, our analyses were consistent with previous research showing that Aβ plaque burden is not well-correlated with indices of clinical severity. However, consistent with our hypothesis, we observed that in participants with aMCI (a stage of continued Aβ accumulation), global Aβ deposition was more strongly inversely associated with synaptic density in the hippocampus compared to those with dementia (a stage of relative Aβ plateau). This stronger association survived PVC (Supplementary Figure 4) and thus is not driven primarily by atrophy. Secondary exploratory analyses across multiple ROIs and whole brain analyses suggested no broad or consistent associations between global Aβ deposition and regional SV2A in either diagnostic group. The nominal negative associations with right-sided subcortical structures in aMCI but right-sided cortical regions with dementia participants (Fig. 3) in the full FreeSurfer region set are not fully mirrored in the corresponding surface-based analyses (Supplementary Figure 3) and may lack biological plausibility.

Additional exploratory ROI-based analyses of the association of regional Aβ deposition and SV2A binding also revealed no consistent pattern but suggested a nominal inverse association in the dementia group between local Aβ deposition and SV2A binding in medial occipital cortex. A more compelling, larger positive correlation was also observed between local Aβ accumulation and synaptic density in the hippocampus (Table 3; Fig. 4). The latter “paradoxical” positive association (which is also present if the aMCI and dementia groups are combined) may relate to the fact that the hippocampus and other medial temporal lobe structures are atrophied to yield low signal with both PET tracers. However, the fact that this positive association is not impacted by PVC (including in the combined aMCI/dementia sample; Supplementary Table 5) suggests an alternative explanation. A theory that we have recently proposed [47–49] is that in early AD medial temporal lobe structures are degenerating so rapidly that the engine for Aβ production is severely compromised, resulting in low levels of Aβ plaques in this region (Supplementary Table 2). In particular, the rapid early destruction of the entorhinal cortex may contribute to reduced Aβ production by neurons that project to hippocampus. Thus, SV2A binding in the hippocampus may represent an index of viable perforant pathway neurons still capable of Aβ production and release, and thus correlates with [^11^C] PiB binding in this region in AD.

### Comparison to previous human studies of Aβ deposition

Our overall results are consistent with prior evidence that Aβ plaques are not well-correlated with indices of disease severity—at least in the dementia stage [27–29]—suggesting a dynamic balance between Aβ deposition and clearance [28, 29]. They are also consistent with evidence from longitudinal PET studies that Aβ deposition eventually approaches a plateau [30]. However, our finding that global Aβ deposition was more strongly inversely associated with synaptic density in the hippocampus in participants with aMCI compared to those with dementia is also consistent with longitudinal PET studies. Such studies have demonstrated continued accumulation through the prodromal stages [31–33] but with minimal change by the time of conversion to AD dementia [31, 34] or in the dementia stage [35]. Our results are further compatible with models of AD in which Aβ initiates the cascade but later uncouples from neurodegenerative processes [34, 50, 51]. No previous study has examined the relationship between Aβ plaque deposition and synaptic density in vivo. However, a number of studies have examined Aβ deposition in relation to other measures of neurodegeneration, including MRI volumetry [52] and [^18^F]FDG-PET glucose metabolism [53], and have also observed this dissociation between Aβ deposition and neurodegenerative changes.

### Postmortem and in vitro studies of Aβ and synapse density

Although no previous human studies have investigated in vivo the relationship between Aβ accumulation and synaptic density, postmortem studies have provided limited evidence that soluble Aβ species [7] and Aβ oligomers (Aβo) [9] are associated with loss of synapses and synaptic proteins. However, measures of Aβ deposition, Aβ-immunoreactive plaques, thioflavin histofluorescent plaques, and concentrations of insoluble Aβ have not been observed to correlate with synaptic change [7].

Non-human studies conducted in APP/PS1 transgenic mice have more fully explored the localized relationship between insoluble Aβ and synapses, demonstrating a loss of synapses or dendritic spines in proximity to plaques [6, 10, 11]. These studies have also suggested a halo of neurotoxic soluble Aβo surrounding senile plaques, resulting in up to 60% loss of excitatory synapses within 50 μm of the plaque [6]. Therefore, fibrillar Aβ may act as a local reservoir of neurotoxic Aβo which results in the loss of proximal dendritic spines and synapses [6]. In the human brain, synaptic density has been observed to decrease progressively as the proximity to senile plaques increase, from normal levels of synaptic density at distances > 50 μm away from the nearest plaque to a ~ 65% reduction in synaptic density levels near the edge of plaques (in association with Aβo concentrations) [8]. Our exploratory ROI-based analyses of the association of regional Aβ deposition and SV2A binding revealed no consistent pattern and therefore cannot lend support to this model of fibrillar Aβ (such as [^11^C] PiB is capable of measuring) acting as a local reservoir of synaptotoxic Aβo. However, our methods do not permit us to address highly localized (< 50 μm) associations.

### Limitations

Important limitations of this study include the small sample size (aMCI = 14, dementia = 24) that hindered us from detecting regionally specific associations between Aβ deposition and synaptic density across diagnostic groups. The other major limitation is the absence of longitudinal data that would enable us to study disease stage-specific relationships between Aβ deposition and synaptic density within participants. In particular, although we observed that in participants with aMCI, global Aβ deposition was more strongly inversely associated with synaptic density in the hippocampus compared to those with dementia, we found no significant differences in mean Aβ deposition ([^11^C] PiB *DVR*) between participants with aMCI and mild dementia (Supplementary Table 2). The latter finding may appear inconsistent with the notion that aMCI is a stage of continued Aβ accumulation, while dementia is a stage of relative Aβ plateau. This discrepancy may reflect the large inter-individual variability in Aβ deposition that is present even at similar disease stages [30–34]. However, longitudinal studies would enable us to define stages of disease, including periods of Aβ accumulation and plateau, within participants and to analyze the relationship between longitudinal changes in Aβ deposition and synaptic loss.

### Conclusions and future directions

To our knowledge, we have conducted the first in vivo study investigating the relationship between Aβ deposition and synaptic alterations in participants with AD. We observed significant inverse associations between measures of global Aβ deposition and hippocampal synaptic density within participants with aMCI but not with dementia. Our findings lend support to a model in which Aβ is still accumulating in the early stages of clinical disease but approaching a relative plateau as a pool of primarily insoluble fibrillar Aβ, a point at which Aβ may uncouple from neurodegenerative processes including synaptic loss. Future research should investigate the relationship between Aβ deposition and synaptic loss in larger cohorts (beginning at preclinical stages of AD) followed longitudinally in conjunction with other markers of pathogenesis.

## Supplementary Information

**Additional file 1: Supplementary Methods.**

**Additional file 2: Supplementary Figures and Tables. ** Supplementary Figure 1. Correlation of *DVR* from 1TC modeling vs. SRTM2 with a cerebellar or CS reference region. Supplementary Table 1. Synaptic density in regions of interest. Supplementary Table 2. Aβ deposition in regions of interest. Supplementary Table 3. Gray matter volume in regions of interest. Supplementary Figure 2. Comparison of SV2A and Aβ deposition in CN, aMCI, and dementia groups after IY-PVC. Supplementary Figure 3. Surface-based, whole-cortex correlations between global Aβ burden and SV2A in aMCI and dementia. Supplementary Figure 4. Correlation of global Aβ deposition and hippocampal SV2A in aMCI and dementia after IY-PVC. Supplementary Table 4. Correlation of global Aβ deposition and regional SV2A in aMCI and dementia after IY-PVC.: Supplementary Figure 5. Brain maps of correlations between global Aβ deposition and SV2A after IY-PVC. Supplementary Table 5. Correlation of regional Aβ deposition and regional SV2A in aMCI and dementia after IY-PVC. Supplementary Figure 6. Brain maps of correlations between regional Aβ deposition and SV2A after IY-PVC.

## Data Availability

The datasets used and/or analyzed in the current study are not publicly available but are available from the corresponding author on reasonable request. In addition, detailed methods and statistical analyses are made available in Additional file 1, while Supplementary Figures and Tables are made available in Additional file 2.

## Abbreviations

Aβ: Amyloid-β
AD: Alzheimer’s disease
*BP*_ND_: Binding potential non-displaceable
Cb: Cerebellum
CDR: Clinical Dementia Rating Scale
CDR-SB: CDR sum of boxes
CN: Cognitively normal
CS: Centrum semiovale
*DVR*: Distribution volume ratio
*DVR*_Cb_: Distribution volume ratio using a cerebellum reference region
*DVR*_CS_: Distribution volume ratio using a centrum semiovale reference region
GDS: Geriatric depression scale
GM: Gray matter
IY: Iterative Yang
LMII: Logical Memory II
aMCI: Amnestic mild cognitive impairment
MMSE: Mini-Mental Status Exam
MRI: Magnetic resonance imaging
PCC: Posterior cingulate cortex
PET: Positron emission tomography
PVC: Partial volume correction
RAVLT: Rey Auditory Verbal Learning Test
SD: Standard deviation
ROI: Region of interest
SRTM2: Simplified reference tissue model—2 step
SV2A: Synaptic vesicle glycoprotein 2A
UPSIT: University of Pennsylvania Smell Identification Test
*V*_T_: Volume of distribution (total)
1TC: 1 tissue compartment
PiB: Pittsburgh Compound B
