# Supplementary material for: Association of Aβ deposition and regional synaptic density in early Alzheimer’s disease: a PET imaging study with [11C]UCB-J
Source: Alzheimers Res Ther. 2021 Jan 5;13:11. doi: 10.1186/s13195-020-00742-y (PMC7786921; doi:10.1186/s13195-020-00742-y)
Supplement: Supplementary file 1 — Additional file 1: Supplementary Methods. [file 13195_2020_742_MOESM1_ESM.docx]

**SUPPLEMENTARY METHODS**

**Association of Aβ deposition and regional synaptic density in early Alzheimer’s disease: a PET imaging study with [^11^C]UCB-J**

Ryan S. O’Dell, MD, PhD,^a,b^ Adam P. Mecca, MD, PhD,^a,b^ Ming-Kai Chen, MD,^c^ PhD,^e^ Mika Naganawa, PhD,^c^ Takuya Toyonaga, MD, PhD,^c^ Yihuan Lu, PhD,^c^ Tyler A. Godek,^a,b^ Joanna E. Harris,^a,b^ Hugh H. Bartlett,^a,b^ Emmie R. Banks,^a,b^ Victoria L. Kominek,^a,b^ Wenzhen Zhao,^a,b^ Nabeel B. Nabulsi, PhD,^c^ Jim Ropchan, PhD,^C^ Yunpeng Ye, PhD,^c^ Brent C. Vander Wyk, PhD,^d^ Yiyun Huang, PhD,^c^ Amy F. T. Arnsten, PhD,^e^ Richard E. Carson, PhD,^c^ and Christopher H. van Dyck, MD^a,b,e,f^

^a^Alzheimer’s Disease Research Unit, Yale University School of Medicine, One Church Street, 8^th^ Floor, New Haven, CT, 06510, USA

^b^Department of Psychiatry, Yale University School of Medicine, 300 George Street, New Haven, CT, 06510, USA

^c^Department of Radiology and Biomedical Imaging, Yale University School of Medicine, P.O. Box 208048, New Haven, CT, 06520, USA

^d^Program on Aging, Yale University School of Medicine, P.O. Box 207900, New Haven, CT, 06520, USA

^e^Department of Neuroscience, Yale University School of Medicine, P.O. Box 208001, New Haven, CT, 06520, USA

^f^Department of Neurology, Yale University School of Medicine, P.O. Box 208018, New Haven, CT, 06520, USA

For correspondence or reprints contact:

Christopher van Dyck, M.D.

Alzheimer’s Disease Research Unit

Yale University School of Medicine

One Church Street, 8^th^ Floor

New Haven, CT 06510

tel +1 203 764-8100

fax +1 203 764-8111

Email: [christopher.vandyck@yale.edu](mailto:christopher.vandyck@yale.edu)

**Table of Contents:**

| **Content** | **Pages** |
| --- | --- |
| Study participants and design | 3 |
| Magnetic resonance imaging | 3 - 4 |
| Partial volume correction | 4 |
| Surface-based vertex-wise analysis | 4 |
| Validation of conversion from centrum semiovale to cerebellar reference region in [^11^C]UCB-J PET | 5 |
| Statistical analyses: Comparison of Aβ deposition and synaptic density across diagnostic groups | 5 - 6 |
| Statistical analyses: Association of Aβ deposition and synaptic density | 6 - 8 |
| References | 8 - 9 |

***Study participants and design***

All participants received a positron emission tomography (PET) scan with [^11^C]Pittsburgh Compound B ([^11^C]PiB) to determine the presence of brain Aβ accumulation as previously described (1-3). The [^11^C]PiB PET scan was determined positive if both visual and quantitative criteria were met. Visual criteria were evaluated by an experienced reader (A.P.M), and quantitative criteria required a [^11^C]PiB cerebral-to-cerebellar distribution volume ratio (*DVR*) of 1.40 or more in at least 1 AD-affected region of interest (ROI)(2, 3). Using these criteria, all participants with dementia and aMCI were determined to be Aβ+ and all CN participants Aβ-.

***Magnetic resonance imaging***

Magnetic resonance imaging (MRI) was performed on a 3T Trio (Siemens Medical Systems, Erlangen, Germany) with a circularly polarized head coil. MR acquisition consisted of a Sag 3D magnetization-prepared rapid gradient-echo (MPRAGE) sequence with 3.34-msec echo time, 2500-msec repetition time, 1100-msec inversion time, 7º flip angle, and 180 Hz/pixel bandwidth. Images are 256x256x176 with a pixel size of 0.98x0.98x1.0 mm. MRI was performed to ensure participants did not show evidence of infection, infarction, or other brain lesions, and was also used to define anatomy, to evaluate atrophy, and to perform partial volume correction (PVC)(4).

For each participant’s MRI, cortical reconstruction and volumetric segmentation was performed using FreeSurfer [version 6.0, http://surfer.nmr.mhg.harvard.edu/](5). Regions from the FreeSurfer segmentation were used for both PET and MRI analyses performed in native subject space, with cortical regions defined by the Desikan-Killiany atlas (6). We have previously published a list of FreeSurfer regions contained within larger ROIs used for analyses (3).

***Partial volume correction***

In addition to analysis of uncorrected data, PVC was performed using the Iterative Yang (IY) algorithm, according to previously described procedures (7, 8). IY provides a voxel-based PVC image and corrects all the FreeSurfer segmented regions for partial volume effects. In IY, the correction map for each voxel is updated iteratively and the final correction map is then applied to the original PET image to perform voxel-based PVC. We used 15 iterations for [^11^C]PiB and 10 iterations for [^11^C]UCB-J due to slightly different convergence behavior. A 3-mm full width half maximum (FWHM) Gaussian kernel was used (9) and the IY-PVC method was applied to each dynamic frame, which was then resliced into the MR space of each participant.

***Surface-based vertex-wise analysis***

For surface-based correlations between Aβ deposition and synaptic density, parametric images of [^11^C]PiB and [^11^C]UCB-J were co-registered to native subject space and sampled onto the cortical surface by averaging *DVR* values across the middle 80% of the cortical ribbon in 10% increments. Surface maps were then transformed to a common (fsaverage) space, and a spatial smooth with a 10 mm FWHM Gaussian kernel was applied prior to statistical analysis on a vertex-wise level using a custom Matlab script. After sampling to the cortical surface, vertex-wise correlations between SV2A binding and global Aβ deposition were also performed with general linear models using FreeSurfer. Permutation was used to correct for multiple comparisons. The cluster-forming threshold was *P* < 0.01 and the cluster-wise threshold was *P* < 0.05.

***Validation of conversion from centrum semiovale to cerebellar reference region in [^11^C]UCB-J PET***

A sample consisting of 51 CN participants, 5 aMCI participants, and 6 dementia participants with arterial blood sampling was used to validate the conversion of *BP*_ND_ with a centrum semiovale (CS) reference region to values of *DVR* with a cerebellar reference region. The 1-tissue compartment (1TC) model was used to calculate *DVR* with a whole cerebellum reference region, in that *DVR* = *V_T_*[ROI]/*V_T_*[cerebellum]. In addition, *BP*_ND_ was calculated using SRTM2 from 0-60 min with either a CS or whole cerebellum reference region. Using the CS reference region, *DVR* was calculated by conversion as *DVR* = (*BP*_ND_[ROI] +1)/(*BP*_ND_[cerebellum]+1). Correlations between *DVR* obtained directly from the SRTM2 kinetic model using a whole cerebellum reference region and those computed using the 1TC model and metabolite-corrected arterial plasma curves, as well as those converted from *BP*_ND_ using a CS reference region were preformed (**Supplementary Figure 1**).

***Statistical analyses***

Statistical analyses were performed using SPSS version 21.0 (IBM Corp.), MATLAB R2019b Statistics Toolbox (Mathworks, Inc.), and FreeSurfer [version 6.0].

***Comparison of Aβ deposition and synaptic density across diagnostic groups***

Separate linear mixed models were used to compare [^11^C]PiB *DVR* and [^11^C]UCB-J *DVR* across multiple ROIs between CN, aMCI, and dementia groups. [^11^C]PiB *DVR* or [^11^C]UCB-J *DVR* was the dependent variable, ROI was included as a repeated measure, and diagnostic group (CN, aMCI, or dementia) and ROI were included as independent variables. The best-fitting variance-covariance structure was compound symmetry as determined by the Bayesian information criterion. Post-hoc tests included ANOVAs within each ROI followed by unpaired t-tests for between group comparisons within an ROI. The Benjamini-Hochberg procedure was used to control the false discovery rate (FDR) for multiple comparisons (12 comparisons for ROIs, and 3 comparisons for diagnostic groups). Analyses of group differences in brain Aβ deposition and synaptic density were performed for medial temporal (entorhinal, hippocampus, parahippocampal, amygdala), prefrontal, lateral temporal, posterior cingulate/precuneus, anterior cingulate, lateral parietal, lateral occipital, medial occipital, and pericentral ROIs, as previously described (3). For display, parametric images of *DVR* for [^11^C]PiB and [^11^C]UCB-J were averaged across participant groups, co-registered to a common MNI template, and overlaid on an MNI template T1 MRI.

***Association of Aβ deposition and synaptic density***

The primary analysis of the association between global Aβ deposition and hippocampal synaptic density in participants with aMCI and dementia was investigated via the use of separate univariate regression analyses for each diagnostic group with correlation coefficients (Pearson *r*) and associated two-tailed *P* values reported for each model. For sensitivity analyses, separate multiple linear regression models were fit that also included covariates of age and sex. Fisher *z*-transformation was used to assess for significant differences in correlation coefficients between the aMCI and dementia groups, with one-tailed *P* values reported, based on the primary assumption of stronger correlation in aMCI than mild dementia. Global Aβ deposition was determined for a composite of regions commonly affected by Aβ deposition in AD which included: prefrontal, lateral temporal, posterior cingulate/precuneus, and lateral parietal ROIs.

Exploratory analyses of the association between global Aβ deposition and regional synaptic density, as well as regional Aβ deposition and regional synaptic density in aMCI and dementia groups were investigated using Pearson’s correlation. Fisher *z*-transformation was used to determine between-group differences in correlation coefficients, with one-tailed *P* values reported. As these analyses were exploratory, corrections for multiple comparisons were not performed. For display, brain maps of correlations between global Aβ deposition and regional synaptic density, as well as between regional Aβ deposition and regional synaptic density were created by producing images with the voxels in each FreeSurfer region set uniformly to the calculated Pearson *r* for that region, overlaid on an MNI template TI MRI and masked to include only significant (*P* < 0.05) correlations.

After sampling to the cortical surface and applying spatial smoothing (described above in ***Surface-based vertex-wise analysis***), surface-based correlations between synaptic density and Aβ deposition (Pearson *r*) were calculated on a vertex-wise level using a custom Matlab script in the aMCI and dementia groups. For display, maps of intra-regional surface-based correlations between *DVR* for [^11^C]PiB and [^11^C]UCB-J were created by producing surface maps (in fsaverage space) with Pearson *r* values mapped to each vertex and masked to include only significant (*P* < 0.05) correlations. In addition, after sampling to the cortical surface and application of a 10 mm spatial smooth, surface-based correlations between predetermined *DVR* measures of global [^11^C]PiB binding and vertex-wise [^11^C]UCB-J binding were calculated using a custom Matlab script, again restricted to the aMCI and dementia groups.

For all of the primary and exploratory analyses described above, similar analyses were preformed using *DVR* from parametric images of [^11^C]PiB and [^11^C]UCB-J that had been partial volume corrected using IY-PVC, as described above in ***Partial volume correction***.

***References***

1. Reiman E, Chen K, Liu X, Bandy D, Yu M, Lee W, et al. Fibrillar amyloid-{beta} burden in cognitively normal people at 3 levels of genetic risk for Alzheimer's disease. Proc Natl Acad Sci U S A. 2009;106(16):6820-5.

2. Mecca AP, Barcelos NM, Wang S, Bruck A, Nabulsi N, Planeta-Wilson B, et al. Cortical beta-amyloid burden, gray matter, and memory in adults at varying APOE epsilon4 risk for Alzheimer's disease. Neurobiol Aging. 2017;61:207-14.

3. Mecca AP, Chen MK, O'Dell RS, Naganawa M, Toyonaga T, Godek TA, et al. In vivo measurement of widespread synaptic loss in Alzheimer's disease with SV2A PET. Alzheimer's & dementia : the journal of the Alzheimer's Association. 2020.

4. Chen MK, Mecca AP, Naganawa M, Finnema SJ, Toyonaga T, Lin SF, et al. Assessing Synaptic Density in Alzheimer Disease With Synaptic Vesicle Glycoprotein 2A Positron Emission Tomographic Imaging. JAMA neurology. 2018;75(10):1215-24.

5. Fischl B. FreeSurfer. Neuroimage. 2012;62(2):774-81.

6. Desikan RS, Segonne F, Fischl B, Quinn BT, Dickerson BC, Blacker D, et al. An automated labeling system for subdividing the human cerebral cortex on MRI scans into gyral based regions of interest. Neuroimage. 2006;31(3):968-80.

7. Erlandsson K, Buvat I, Pretorius PH, Thomas BA, Hutton BF. A review of partial volume correction techniques for emission tomography and their applications in neurology, cardiology and oncology. Phys Med Biol. 2012;57(21):R119-59.

8. Shidahara M, Thomas BA, Okamura N, Ibaraki M, Matsubara K, Oyama S, et al. A comparison of five partial volume correction methods for Tau and Amyloid PET imaging with [18F]THK5351 and [11C]PIB. Ann Nucl Med. 2017;31(7):563-9.

9. Lu Y, Toyonaga T, Naganawa M, Gallezot J-D, Chen M-K, Mecca A, et al. Partial volume correction for PET synaptic density imaging with 11C-UCB-J. Journal of Nuclear Medicine. 2018;59(supplement 1):77.
