# Supplementary material for: Association of Aβ deposition and regional synaptic density in early Alzheimer’s disease: a PET imaging study with [11C]UCB-J
Source: Alzheimers Res Ther. 2021 Jan 5;13:11. doi: 10.1186/s13195-020-00742-y (PMC7786921; doi:10.1186/s13195-020-00742-y)
Supplement: Supplementary file 2 — Additional file 2: Supplementary Figures and Tables. Supplementary Figure 1. Correlation of DVR from 1TC modeling vs. SRTM2 with a cerebellar or CS reference region. Supplementary Table 1. Synaptic density in regions of interest. Supplementary Table 2. Aβ deposition in regions of interest. Supplementary Table 3. Gray matter volume in regions of interest. Supplementary Figure 2. Comparison of SV2A and Aβ deposition in CN, aMCI, and dementia groups after IY-PVC. Supplementary Figure 3. Surface-based, whole-cortex correlations between global Aβ burden and SV2A in aMCI and dementia. Supplementary Figure 4. Correlation of global Aβ deposition and hippocampal SV2A in aMCI and dementia after IY-PVC. Supplementary Table 4. Correlation of global Aβ deposition and regional SV2A in aMCI and dementia after IY-PVC.: Supplementary Figure 5. Brain maps of correlations between global Aβ deposition and SV2A after IY-PVC. Supplementary Table 5. Correlation of regional Aβ deposition and regional SV2A in aMCI and dementia after IY-PVC. Supplementary Figure 6. Brain maps of correlations between regional Aβ deposition and SV2A after IY-PVC. [file 13195_2020_742_MOESM2_ESM.docx]

**SUPPLEMENTARY FIGURES AND TABLES**

**Association of Aβ deposition and regional synaptic density in early Alzheimer’s disease: a PET imaging study with [^11^C]UCB-J**

Ryan S. O’Dell, MD, PhD,^a,b^ Adam P. Mecca, MD, PhD,^a,b^ Ming-Kai Chen, MD,^c^ PhD,^e^ Mika Naganawa, PhD,^c^ Takuya Toyonaga, MD, PhD,^c^ Yihuan Lu, PhD,^c^ Tyler A. Godek,^a,b^ Joanna E. Harris,^a,b^ Hugh H. Bartlett,^a,b^ Emmie R. Banks,^a,b^ Victoria L. Kominek,^a,b^ Wenzhen Zhao,^a,b^ Nabeel B. Nabulsi, PhD,^c^ Jim Ropchan, PhD,^C^ Yunpeng Ye, PhD,^c^ Brent C. Vander Wyk, PhD,^d^ Yiyun Huang, PhD,^c^ Amy F. T. Arnsten, PhD,^e^ Richard E. Carson, PhD,^c^ and Christopher H. van Dyck, MD^a,b,e,f^

^a^Alzheimer’s Disease Research Unit, Yale University School of Medicine, One Church Street, 8^th^ Floor, New Haven, CT, 06510, USA

^b^Department of Psychiatry, Yale University School of Medicine, 300 George Street, New Haven, CT, 06510, USA

^c^Department of Radiology and Biomedical Imaging, Yale University School of Medicine, P.O. Box 208048, New Haven, CT, 06520, USA

^d^Program on Aging, Yale University School of Medicine, P.O. Box 207900, New Haven, CT, 06520, USA

^e^Department of Neuroscience, Yale University School of Medicine, P.O. Box 208001, New Haven, CT, 06520, USA

^f^Department of Neurology, Yale University School of Medicine, P.O. Box 208018, New Haven, CT, 06520, USA

For correspondence or reprints contact:

Christopher van Dyck, M.D.

Alzheimer’s Disease Research Unit

Yale University School of Medicine

One Church Street, 8^th^ Floor

New Haven, CT 06510

tel +1 203 764-8100

fax +1 203 764-8111

Email: christopher.vandyck@yale.edu

**Table of Contents:**

| **Content** | **Pages** |
| --- | --- |
| Supplementary Figure 1. Correlation of *DVR* from 1TC modeling vs. SRTM2 with a cerebellar or CS reference region | 3 - 4 |
| Supplementary Table 1. Synaptic density in regions of interest | 5 |
| Supplementary Table 2. Aβ deposition in regions of interest | 6 |
| Supplementary Table 3. Gray matter volume in regions of interest | 7 - 8 |
| Supplementary Figure 2. Comparison of SV2A and Aβ deposition in CN, aMCI, and dementia groups after IY-PVC | 9 - 10 |
| Supplementary Figure 3. Surface-based, whole-cortex correlations between global Aβ burden and SV2A in aMCI and dementia | 11 - 12 |
| Supplementary Figure 4. Correlation of global Aβ deposition and hippocampal SV2A in aMCI and dementia after IY-PVC | 13 -14 |
| Supplementary Table 4. Correlation of global Aβ deposition and regional SV2A in aMCI and dementia after IY-PVC | 15 - 16 |
| Supplementary Figure 5. Brain maps of correlations between global Aβ deposition and SV2A after IY-PVC | 17 - 18 |
| Supplementary Table 5. Correlation of regional Aβ deposition and regional SV2A in aMCI and dementia after IY-PVC | 19 |
| Supplementary Figure 6. Brain maps of correlations between regional Aβ deposition and SV2A after IY-PVC | 20 - 21 |

**
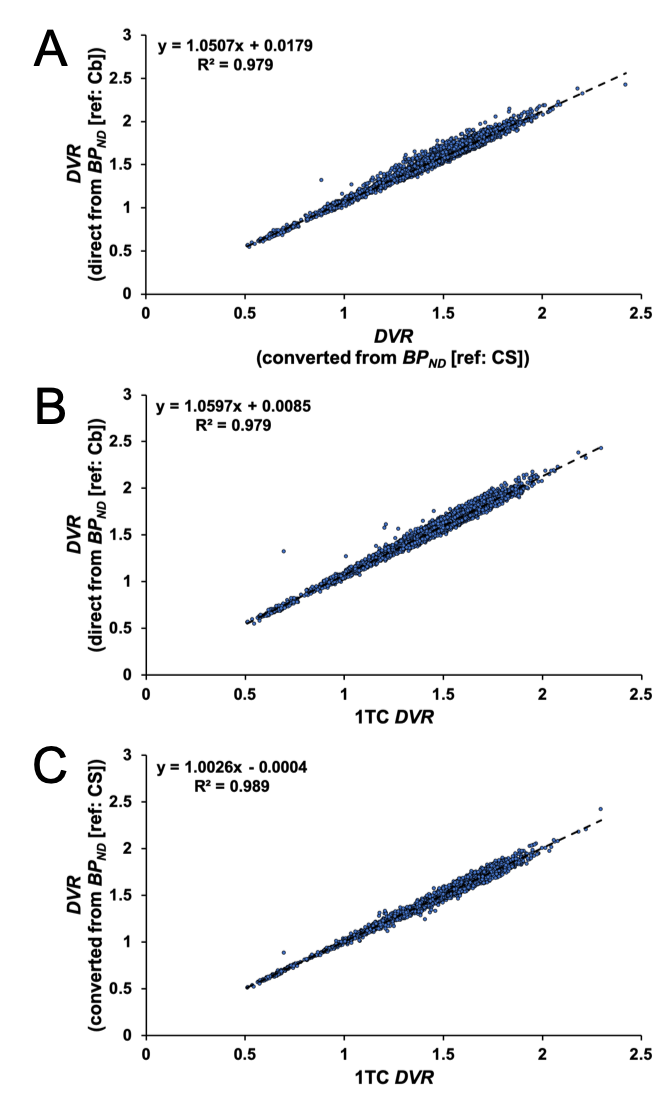
**

**Supplementary Figure 1. Correlation of *DVR* from 1TC modeling vs. SRTM2 with a cerebellar or CS reference region.** In this sample of 51 CN, 5 aMCI, and 6 dementia participants, regional values of [^11^C]UCB-J *DVR* with a cerebellar reference region were directly calculated using the SRTM2 kinetic model from 0-60 minutes, with a new population average *k*_2_ of the whole cerebellum using the 1TC model ($k_{2}^{'}$= 0.023 min^-1^). (A) Values of *DVR* calculated directly with a cerebellar reference region were highly correlated across all FreeSurfer brain regions with (A) values of *DVR* converted from *BP*_ND_ with a CS reference region (*r* = 0.989, *P* < 0.0001). In addition, (B) values of *DVR* calculated directly with a cerebellar reference region were highly correlated with *DVR* obtained using the 1TC model and metabolite-corrected arterial plasma curves and a cerebellar reference region (*r* = 0.989, *P* < 0.0001). Lastly, (C) values of *DVR* converted from *BP*_ND_ with a CS reference region also demonstrated strong correlations with values of *DVR* obtained using the 1TC model and metabolite-corrected arterial plasma curves and cerebellar reference region across all FreeSurfer brain regions (*r* = 0.994, *P* < 0.0001). Abbreviations: *DVR*, distribution volume ratio using a cerebellum reference region; 1TC, 1 tissue compartment; *BP_ND_*, binding potential non-displaceable; SRTM2, simplified reference tissue model – 2 step; CS, centrum semiovale; Cb, cerebellum; CN, cognitively normal; AD, Alzheimer’s disease; aMCI, amnestic mild cognitive impairment; *V_T_*, volume of distribution; ROI, region of interest.

| **Supplementary Table 1. Synaptic density in regions of interest** | | | | | | | | |
| --- | --- | --- | --- | --- | --- | --- | --- | --- |
|  | **[^11^C]UCB-J *DVR*** | | | | **[^11^C]UCB-J *DVR* IY-PVC** | | | |
| **Region** | **CN** | **aMCI** | **Dementia** | **One Way ANOVA** | **CN** | **aMCI** | **Dementia** | **One Way ANOVA** |
| **Entorhinal** | 1.20 (0.11) | 1.01 (0.11)** | 1.04 (0.14)** | F(2,54)=11.64  *P*=0.0004 | 1.27 (0.13) | 1.07 (0.13)** | 1.11  (0.16)** | F(2,54)=9.61  *P*=0.002 |
| **Hippocampus** | 1.04 (0.09) | 0.87 (0.12)** | 0.87 (0.12)*** | F(2,54)=14.11  *P*=0.0001 | 1.11 (0.14) | 0.90 (0.14)** | 0.90  (0.13)*** | F(2,54)=14.35  *P*=0.0001 |
| **Parahippocampal** | 1.24 (0.09) | 1.14 (0.10)* | 1.13 (0.13)* | F(2,54)=5.91  *P*=0.01 | 1.34 (0.10) | 1.24 (0.14) | 1.23 (0.16)* | F(2,54)=4.11  *P*=0.07 |
| **Amygdala** | 1.36 (0.12) | 1.19 (0.20)* | 1.28  (0.19) | F(2,54)=3.69  *P*=0.04 | 1.47 (0.14) | 1.30 (0.22) | 1.40  (0.21) | F(2,54)=2,91  *P*=0.13 |
| **Lateral Temporal** | 1.59 (0.08) | 1.48 (0.14)* | 1.43 (0.12)*** | F(2,54)=10.42  *P*=0.0006 | 1.84 (0.13) | 1.75 (0.17) | 1.67 (0.15)* | F(2,54)=6.16  *P*=0.02 |
| **Prefrontal** | 1.59 (0.09) | 1.52 (0.13) | 1.49 (0.10)* | F(2.54)=5.60  *P*=0.01 | 1.84 (0.16) | 1.79 (0.17) | 1.75  (0.13) | F(2,54)=1.84  *P*=0.20 |
| **PCC/Precuneus** | 1.58 (0.07) | 1.49 (0.12) | 1.44 (0.15)* | F(2,54)=7.10  *P*=0.004 | 1.79 (0.12) | 1.75 (0.15) | 1.68  (0.19) | F(2,54)=2.53  *P*=0.15 |
| **Anterior Cingulate** | 1.63 (0.11) | 1.55 (0.15) | 1.55  (0.14) | F(2,54)=2.40  *P*=0.11 | 1.89 (0.14) | 1.81 (0.17) | 1.81  (0.20) | F(2,54)=1.32  *P*=0.30 |
| **Lateral Parietal** | 1.64 (0.09) | 1.51 (0.16)* | 1.48 (0.14)** | F(2,54)=8.30  *P*=0.002 | 1.92 (0.15) | 1.81 (0.23) | 1.77  (0.18)* | F(2,54)=3.84  *P*=0.07 |
| **Lateral Occipital** | 1.53 (0.08) | 1.48 (0.16) | 1.43 (0.10)* | F(2,54)=4.32  *P*=0.03 | 1.80 (0.13) | 1.76 (0.23) | 1.70  (0.12) | F(2,54)=2.01  *P*=0.19 |
| **Medial Occipital** | 1.45 (0.07) | 1.43 (0.11) | 1.40  (0.10) | F(2,54)=1.67  *P*=0.20 | 1.68 (0.10) | 1.67 (0.14) | 1.64  (0.13) | F(2,54)=0.62  *P*=0.54 |
| **Pericentral** | 1.43 (0.08) | 1.35 (0.12)* | 1.35 (0.10)* | F(2,54)=3.79  *P*=0.04 | 1.65 (0.10) | 1.56 (0.15) | 1.58  (0.12) | F(2,54)=2.25  *P*=0.17 |
| **Global Cortical** | 1.57 (0.08) | 1.48 (0.12)* | 1.44 (0.10)** | F(2,54)=9.48  *P*=0.0003 | 1.82 (0.13) | 1.75 (0.16) | 1.70 (0.12)* | F(2,54)=4.07  *P*=0.02 |

Data are mean (SD). F and *P* statistics are for one-way ANOVAs, performed within each ROI after a linear mixed model analysis of [^11^C]UCB-J *DVR* across multiple ROIs between CN, aMCI, and dementia groups, both before and after PVC. One-way ANOVA associated *P* values are FDR-corrected for 12 ROIs. Unpaired, post-hoc *t*-tests to determine group differences are FDR-corrected for multiple comparisons for 3 diagnostic groups. * denotes significant group differences between either aMCI and CN or dementia and CN. * *P* < 0.05, ** *P* < 0.001, *** *P* < 0.0001. † denotes significant group differences between aMCI and dementia. Abbreviations: *DVR*, distribution volume ratio using a whole cerebellum reference region; PCC, posterior cingulate cortex; SD, standard deviation; IY-PVC, Iterative Yang Partial Volume Correction; ROI, region of interest; FDR, false discovery rate; SV2A, synaptic vesicle glycoprotein 2A; aMCI, amnestic mild cognitive impairment; CN, cognitively normal.

| **Supplementary Table 2. *Aβ* deposition in regions of interest** | | | | | | | | |
| --- | --- | --- | --- | --- | --- | --- | --- | --- |
|  | **[^11^C]PiB *DVR*** | | | | **[^11^C]PiB *DVR* IY-PVC** | | | |
| **Region** | **CN** | **aMCI** | **Dementia** | **One Way ANOVA** | **CN** | **aMCI** | **Dementia** | **One Way ANOVA** |
| **Entorhinal** | 1.00 (0.05) | 1.18 (0.13)*** | 1.18 (0.14)*** | F(2,54)=16.70  *P*<0.0001 | 1.00 (0.07) | 1.12 (0.13)* | 1.13 (0.14)* | F(2,54)=8.11  *P*=0.0009 |
| **Hippocampus** | 1.06 (0.06) | 1.10 (0.12) | 1.06  (0.11) | F(2,54)=0.86  *P*=0.43 | 1.03 (0.05) | 1.04 (0.12) | 1.00  (0.11) | F(2,54)=0.71  *P*=0.50 |
| **Parahippocampal** | 1.06 (0.04) | 1.38 (0.18)*** | 1.40 (0.16)*** | F(2,54)=37.18  *P*<0.0001 | 1.05 (0.05) | 1.37 (0.19)*** | 1.41 (0.18)*** | F(2,54)=33.40  *P*<0.0001 |
| **Amygdala** | 1.11 (0.07) | 1.30 (0.13)*** | 1.30 (0.14)*** | F(2,54)=17.25  *P*<0.0001 | 1.12 (0.07) | 1.29 (0.14)** | 1.30 (0.14)*** | F(2,54)=14.06  *P*<0.0001 |
| **Lateral Temporal** | 1.14 (0.06) | 1.81 (0.35)*** | 1.83 (0.23)*** | F(2,54)=55.80  *P*<0.0001 | 1.21 (0.08) | 1.99 (0.42)*** | 2.00 (0.28)*** | F(2,54)=51.64  *P*<0.0001 |
| **Prefrontal** | 1.20 (0.08) | 2.11 (0.46)*** | 2.08 (0.27)*** | F(2,54)=58.05  *P*<0.0001 | 1.32 (0.10) | 2.38 (0.57)*** | 2.35 (0.35)*** | F(2,54)=51.44  *P*<0.0001 |
| **PCC/Precuneus** | 1.20 (0.07) | 2.13 (0.48)*** | 2.18 (0.25)*** | F(2,54)=70.04  *P*<0.0001 | 1.26 (0.08) | 2.38 (0.59)*** | 2.44 (0.32)*** | F(2,54)=66.64  *P*<0.0001 |
| **Anterior Cingulate** | 1.14 (0.07) | 2.00 (0.42)*** | 2.04 (0.27)*** | F(2,54)=65.73  *P*<0.0001 | 1.19 (0.09) | 2.19 (0.49)*** | 2.22 (0.32)*** | F(2,54)=63.52  *P*<0.0001 |
| **Lateral Parietal** | 1.22 (0.08) | 2.03 (0.42)*** | 2.10 (0.26)*** | F(2,54)=63.05  *P*<0.0001 | 1.33 (0.10) | 2.29 (0.53)*** | 2.38 (0.32)*** | F(2,54)=56.95  *P*<0.0001 |
| **Lateral Occipital** | 1.25 (0.08) | 1.65 (0.21)*** | 1.73 (0.22)*** | F(2,54)=39.08  *P*<0.0001 | 1.37 (0.10) | 1.80 (0.24)*** | 1.91 (0.28)*** | F(2,54)=32.91  *P*<0.0001 |
| **Medial Occipital** | 1.14 (0.05) | 1.43 (0.21)*** | 1.46 (0.17)*** | F(2,54)=24.52  *P*<0.0001 | 1.20 (0.06) | 1.48 (0.22)*** | 1.53 (0.20)*** | F(2,54)=20.84  *P*<0.0001 |
| **Pericentral** | 1.22 (0.09) | 1.69 (0.32)*** | 1.69 (0.20)*** | F(2,54)=29.78  *P*<0.0001 | 1.34 (0.11) | 1.85 (0.39)*** | 1.84 (0.24)*** | F(2,54)=24.74  *P*<0.0001 |
| **Global Cortical** | 1.19 (0.07) | 2.01 (0.42)*** | 2.02 (0.24)*** | F(2,54)=62.93  *P*<0.0001 | 1.28 (0.09) | 2.25 (0.51)*** | 2.26 (0.30)*** | F(2,54)=57.44  *P*<0.0001 |

Data are mean (SD). F and *P* statistics are for one-way ANOVAs, performed within each ROI after a linear mixed model analysis of [^11^C]PiB *DVR* across multiple ROIs between CN, aMCI, and dementia groups, both before and after PVC. One-way ANOVA associated *P* values are FDR-corrected for 12 ROIs. Unpaired, post-hoc *t*-tests to determine group differences are FDR-corrected for multiple comparisons for 3 diagnostic groups. * denotes significant group differences between either aMCI and CN or dementia and CN. * *P* < 0.05, ** *P* < 0.001, *** *P* < 0.0001. † denotes significant group differences between aMCI and dementia. Abbreviations: *DVR*, distribution volume ratio using a whole cerebellum reference region; PCC, posterior cingulate cortex; SD, standard deviation; IY-PVC, Iterative Yang Partial Volume Correction; ROI, region of interest; FDR, false discovery rate; Aβ, amyloid beta; PiB, Pittsburgh Compound B; aMCI, amnestic mild cognitive impairment; CN, cognitively normal.

| **Supplementary Table 3. Gray matter volume in regions of interest** | | | | |
| --- | --- | --- | --- | --- |
|  | **Gray Matter Volume (cm^3^)** | | | |
| **Region** | **CN** | **aMCI** | **Dementia** | **One Way ANOVA** |
| **Entorhinal** | 3.96 (0.66) | 2.98 (0.55)** | 3.20 (0.72)** | F(2,54)=10.62  *P*=0.0008 |
| **Hippocampus** | 7.62 (0.90) | 6.35 (0.88)* | 6.56 (1.06)* | F(2,54)=8.83  *P*=0.002 |
| **Parahippocampal** | 3.87 (0.60) | 3.31 (0.46)* | 3.40 (0.56)* | F(2,54)=5.32  *P*=0.02 |
| **Amygdala** | 2.89 (0.42) | 2.22 (0.36)*** | 2.29 (0.44)*** | F(2,54)=14.55  *P*=0.0001 |
| **Lateral Temporal** | 81.99 (7.23) | 73.89 (6.75)* | 74.34 (6.59)* | F(2,54)=8.27  *P*=0.002 |
| **Prefrontal** | 109.90 (10.10) | 104.62 (9.85) | 108.02 (6.22) | F(2,54)=1.53  *P*=0.34 |
| **PCC/Precuneus** | 27.77 (2.50) | 25.75 (3.02)* | 24.62 (2.13)** | F(2,54)=8.49  *P*=0.002 |
| **Anterior Cingulate** | 6.72 (0.60) | 6.68 (0.82) | 6.91 (1.30) | F(2,54)=0.30  *P*=0.81 |
| **Lateral Parietal** | 60.82 (4.41) | 58.24 (5.88) | 56.28 (4.92)* | F(2,54)=4.37  *P*=0.03 |
| **Lateral Occipital** | 21.30 (2.05) | 20.54 (2.06) | 20.43 (2.57) | F(2,54)=0.85  *P*=0.52 |
| **Medial Occipital** | 22.95 (2.95) | 22.65 (3.00) | 22.75 (2.80) | F(2,54)=0.05  *P*=0.95 |
| **Pericentral** | 48.31 (3.62) | 47.24 (4.53) | 49.34 (3.97) | F(2,54)=1.24  *P*=0.40 |
| **Global Cortical** | 326.84 (24.75) | 304.58 (25.61)* | 306.05 (18.42)* | F(2,54)=5.71  *P*=0.006 |

**Supplementary Table 3.** Data are mean (SD). F and *P* statistics are for one-way ANOVAs, performed within each ROI after a linear mixed model analysis of gray matter volume (cm^3^) across multiple ROIs between CN, aMCI, and dementia groups. One-way ANOVA associated *P* values are FDR-corrected for 12 ROIs. Unpaired, post-hoc *t*-tests to determine group differences are FDR-corrected for multiple comparisons for 3 diagnostic groups. * denotes significant group differences between either aMCI and CN or dementia and CN. * *P* < 0.05, ** *P* < 0.001, *** *P* < 0.0001. † denotes significant group differences between aMCI and dementia. Linear mixed model analysis demonstrated significant effects of ROI (F(11,1043.2) = 7703.5, *P* < 0.0001) and group*ROI interaction (F(22,1043.2) = 5.2, *P* = < 0.0001), but not group (F(2,15.6) = 2.6, *P* = 0.12), as predictors of gray matter volume. Abbreviations: PCC, posterior cingulate cortex; SD, standard deviation; ROI, region of interest; FDR, false discovery rate; aMCI, amnestic mild cognitive impairment; CN, cognitively normal.


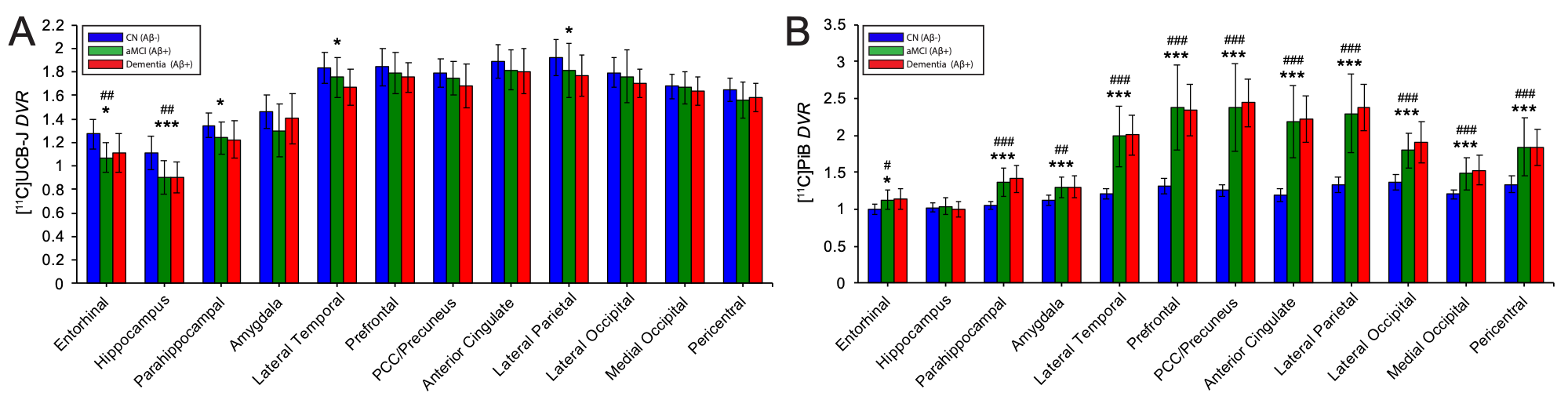


**Supplementary Figure 2. Comparison of SV2A and *Aβ* deposition in CN, aMCI, and dementia groups after IY-PVC.** Quantification of group differences in (A) SV2A binding and (B) Aβ deposition across all ROIs in CN, aMCI, and dementia groups as determined by [^11^C]UCB-J and [^11^C]PiB PET, respectively, after application of IY-PVC. Analyses of [^11^C]UCB-J and [^11^C]PiB *DVR* demonstrated significant effects of group ([^11^C]UCB-J: F(2,54) = 6.2, *P* = 0.004, [^11^C]PiB: F(2,54) = 54.3, *P* < 0.0001), ROI ([^11^C]UCB-J: F(11,594) = 360.8, *P* < 0.0001, [^11^C]PiB: F(11,594) = 280.4, *P* < 0.0001) and group*ROI interaction ([^11^C]UCB-J: F(22,594) = 2.1, *P* = 0.003, [^11^C]PiB: F(22,594) = 40.5, *P* < 0.0001) as predictors of SV2A and Aβ binding. (A) One-way ANOVAs with post-hoc unpaired *t*-tests, FDR-corrected for multiple comparisons (3 comparisons for diagnostic groups), demonstrated significant reductions of [^11^C]UCB-J *DVR* in aMCI and dementia participants in the hippocampus and entorhinal cortex. The parahippocampal, lateral temporal, and lateral parietal cortices demonstrated mildly significant reductions of [^11^C]UCB-J *DVR* in the dementia group, while non-significant trends of SV2A reduction were observed in aMCI participants within these ROIs. (B) Significant elevations of [^11^C]PiB *DVR* in aMCI and dementia participants was observed across all analyzed ROIs, with the exception of the hippocampus. No group differences in SV2A or Aβ binding between aMCI and dementia participants were observed in all analyzed ROIs. # denotes significant post-hoc group differences between aMCI and CN, while * denotes significant group post-hoc differences between dementia and CN. #/* *P* < 0.05, ##/** *P* < 0.001, ###/*** *P* < 0.0001. Abbreviations: IY-PVC, Iterative-Yang Partial Volume Correction; *DVR*, distribution volume ratio using a whole cerebellum reference region; PCC, posterior cingulate cortex, aMCI: amnestic mild cognitive impairment; CN: cognitively normal; PET, positron emission tomography; ROI, region of interest; SV2A, synaptic vesicle glycoprotein 2A; Aβ, amyloid beta; PiB, Pittsburgh Compound B.


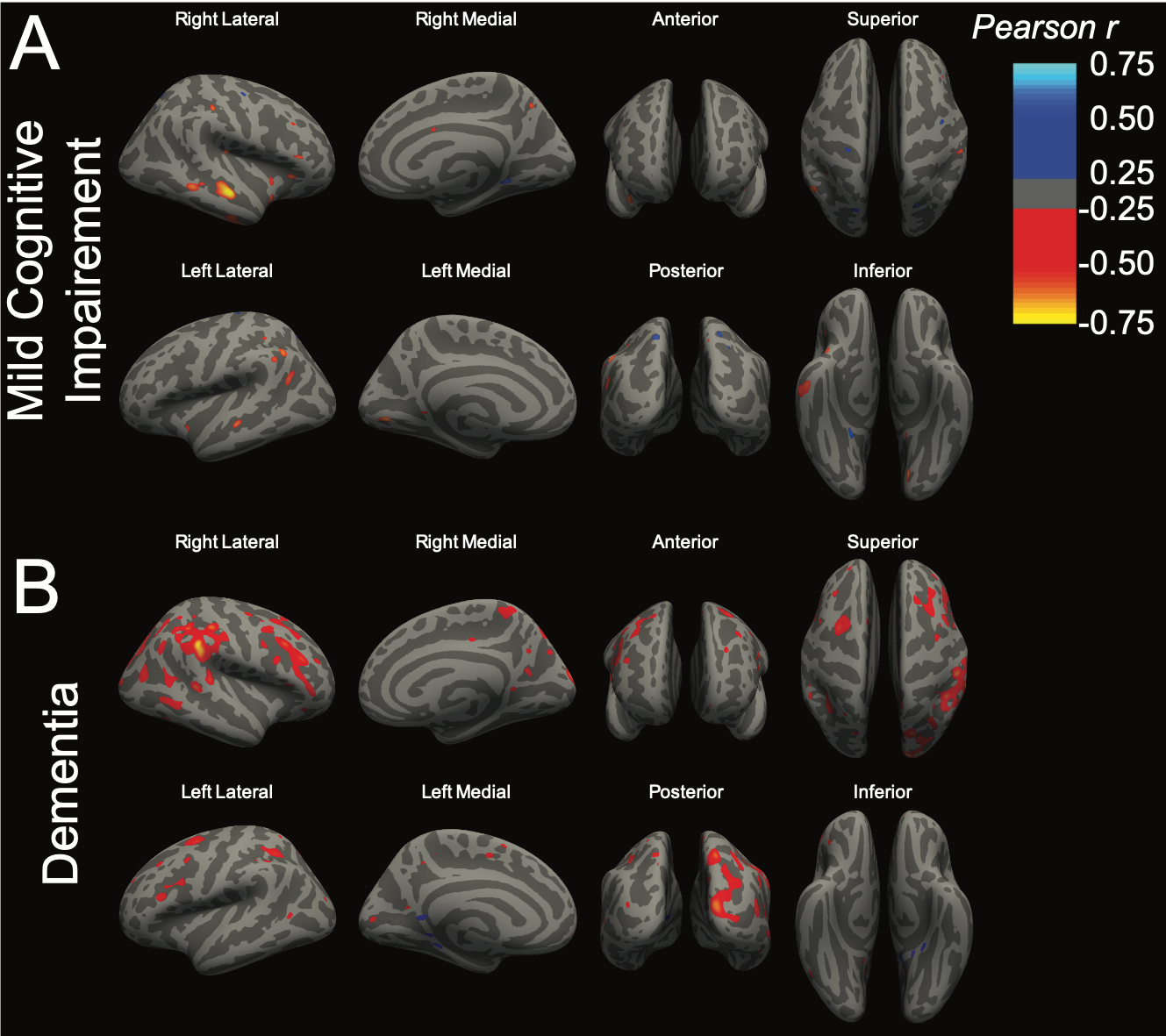


**Supplementary Figure 3. Surface-based, vertex-wise correlations between global *Aβ* burden and SV2A in aMCI and dementia.** [^11^C]UCB-J parametric images were sampled onto the cortical surface by averaging *DVR* values across the middle 80% of the cortical ribbon and then transformed to a common space (fsaverage). A spatial smooth with a 10 mm FWHM Gaussian kernel was applied prior to correlation analysis on a vertex-wise level with previously determined measures of global Aβ deposition using a custom Matlab script. Correlation maps show Pearson *r* values and are thresholded at an uncorrected *P* < 0.05 with both the (A) aMCI and (B) dementia diagnostic groups. MR image slices adhere to radiological convention, with orientation denoted in the first coronal section of each image series. Abbreviations: *DVR*, distribution volume ratio using a whole cerebellum reference region; Aβ, amyloid beta; PiB, Pittsburgh Compound B; aMCI, amnestic mild cognitive impairment.


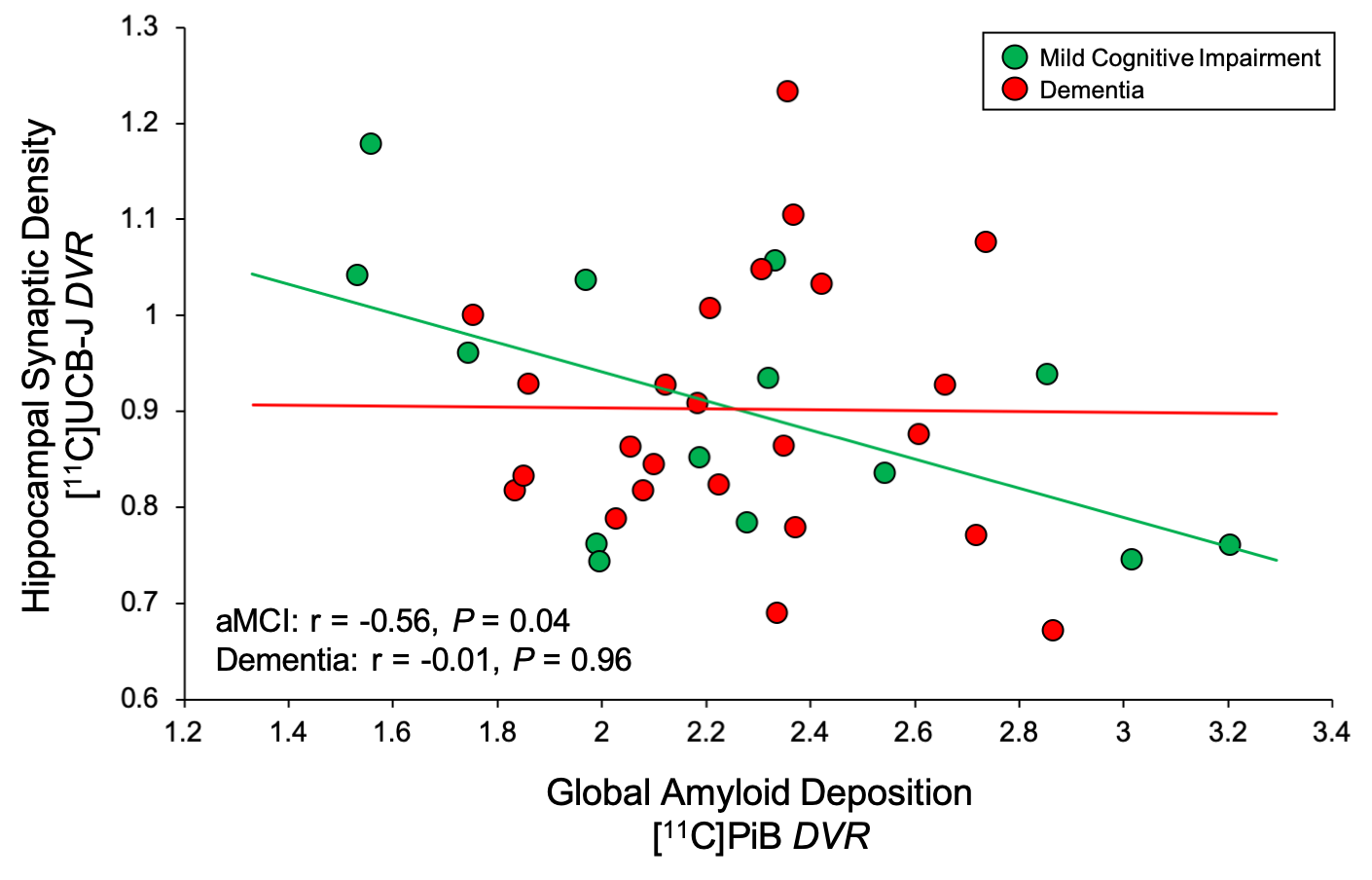


**Supplementary Figure 4. Correlation of global *Aβ* deposition and hippocampal SV2A in aMCI and dementia after IY-PVC.** After application of IY-PVC to [^11^C]UCB-J and [^11^C]PiB *DVR* parametric images, correlation coefficients (Pearson *r*) were calculated from separate univariate linear regression analyses in each group with associated two-tailed *P* values, without correction for multiple comparisons. Similar to non-PVC data, the scatter plot with best-fit lines depicts a significant inverse association between global Aβ deposition and hippocampal SV2A in participants with aMCI (green; *r* = -0.56, *P* = 0.04) but not with dementia (red; *r* = -0.01, *P* = 0.96). The addition of age and sex as covariates to this model mildly reduced the previously observed significance in the aMCI group (R^2^ = 0.39, semi-partial correlation coefficient = -0.49, *P* = 0.08), while the association between global Aβ deposition and hippocampal SV2A in the dementia group remained non-significant (R^2^ = 0.21, semi-partial correlation coefficient = 0.004, *P* = 0.99). Global cortical Aβ deposition was calculated by averaging binding values (*DVR*) from the bilateral prefrontal, lateral temporal, posterior cingulate/precuneus, and lateral parietal ROIs, weighted by volume. Green circles denote *DVR* values for aMCI participants, while red circles denote *DVR* values for participants with dementia. Abbreviations: IY-PVC, Iterative Yang Partial Volume Correction; *DVR*, distribution volume ratio using a whole cerebellum reference region; aMCI: amnestic mild cognitive impairment; SV2A, synaptic vesicle glycoprotein 2A; Aβ, amyloid beta; PiB, Pittsburgh Compound B.

| **Supplementary Table 4. Correlation of global *Aβ* deposition and regional SV2A in aMCI and dementia after IY-PVC** | | | | | | |
| --- | --- | --- | --- | --- | --- | --- |
|  | **Mild Cognitive Impairment (n = 14)** | | **Dementia (n = 24)** | | **Fisher *z*-transform** | |
| **Primary Region** | **Pearson *r*** | ***P*** | **Pearson *r*** | ***P*** | ***z*** | ***P*** |
| **Hippocampus** | -0.56 | 0.04* | -0.01 | 0.96 | -1.67 | 0.047 |
| **Exploratory Regions** | **Pearson *r*** | ***P*** | **Pearson *r*** | ***P*** | ***z*** | ***P*** |
| **Entorhinal** | -0.07 | 0.82 | 0.08 | 0.70 | -0.40 | 0.34 |
| **Parahippocampal** | -0.29 | 0.31 | -0.02 | 0.92 | -0.75 | 0.23 |
| **Amygdala** | -0.44 | 0.11 | 0.03 | 0.87 | -1.35 | 0.09 |
| **Lateral Temporal** | -0.25 | 0.39 | -0.09 | 0.69 | -0.44 | 0.33 |
| **Prefrontal** | 0.02 | 0.94 | -0.07 | 0.74 | 0.24 | 0.40 |
| **PCC/Precuneus** | -0.08 | 0.79 | -0.27 | 0.20 | 0.53 | 0.30 |
| **Anterior Cingulate** | 0.12 | 0.69 | -0.09 | 0.68 | 0.57 | 0.29 |
| **Lateral Parietal** | -0.11 | 0.70 | -0.35 | 0.10 | 0.69 | 0.25 |
| **Lateral Occipital** | -0.07 | 0.81 | -0.28 | 0.19 | 0.59 | 0.28 |
| **Medial Occipital** | -0.16 | 0.58 | -0.18 | 0.41 | 0.06 | 0.48 |
| **Pericentral** | 0.05 | 0.86 | -0.06 | 0.79 | 0.30 | 0.38 |

Data are Pearson *r* and associated two-tailed *P* values obtained from separate univariate linear regression analyses in each group, uncorrected for multiple comparisons. After application of IY-PVC, global cortical Aβ deposition was calculated by averaging binding values ([^11^C]PiB *DVR*) from the bilateral prefrontal, lateral temporal, posterior cingulate/precuneus, and lateral parietal ROIs, weighted by volume. This approximation of global Aβ burden was then correlated with [^11^C]UCB-J *DVR* from the 4 medial temporal structures and 8 neocortical ROIs. To determine significant differences between group correlation coefficients, Fisher *z*-transformations and associated one-tailed *P* values were reported. Similar to non-PVC data, global Aβ deposition was significantly correlated with reductions in hippocampal SV2A binding in participants with aMCI (*r* = -0.56, *P* = 0.04), but not in those with dementia (*r* = -0.01, *P* = 0.96). This difference between group correlation coefficients was significant (Fisher *z* = -1.67, one-tailed *P* = 0.047). In participants with aMCI, no significant correlations between global Aβ deposition and regional SV2A were observed in exploratory ROIs. In addition, the previously demonstrated nominal inverse association between global Aβ deposition and lateral parietal SV2A in the dementia group was not observed after PVC (*r* = -0.35, *P* = 0.10). * denotes significant correlation, with *P* < 0.05. Abbreviations: IY-PVC, Iterative Yang Partial Volume Correction; PCC, posterior cingulate cortex; Aβ, amyloid beta; PiB, Pittsburgh Compound B; SV2A, synaptic vesicle glycoprotein 2A; *DVR*, distribution volume ratio using a whole cerebellum reference region; ROI; region of interest; aMCI, amnestic mild cognitive impairment.


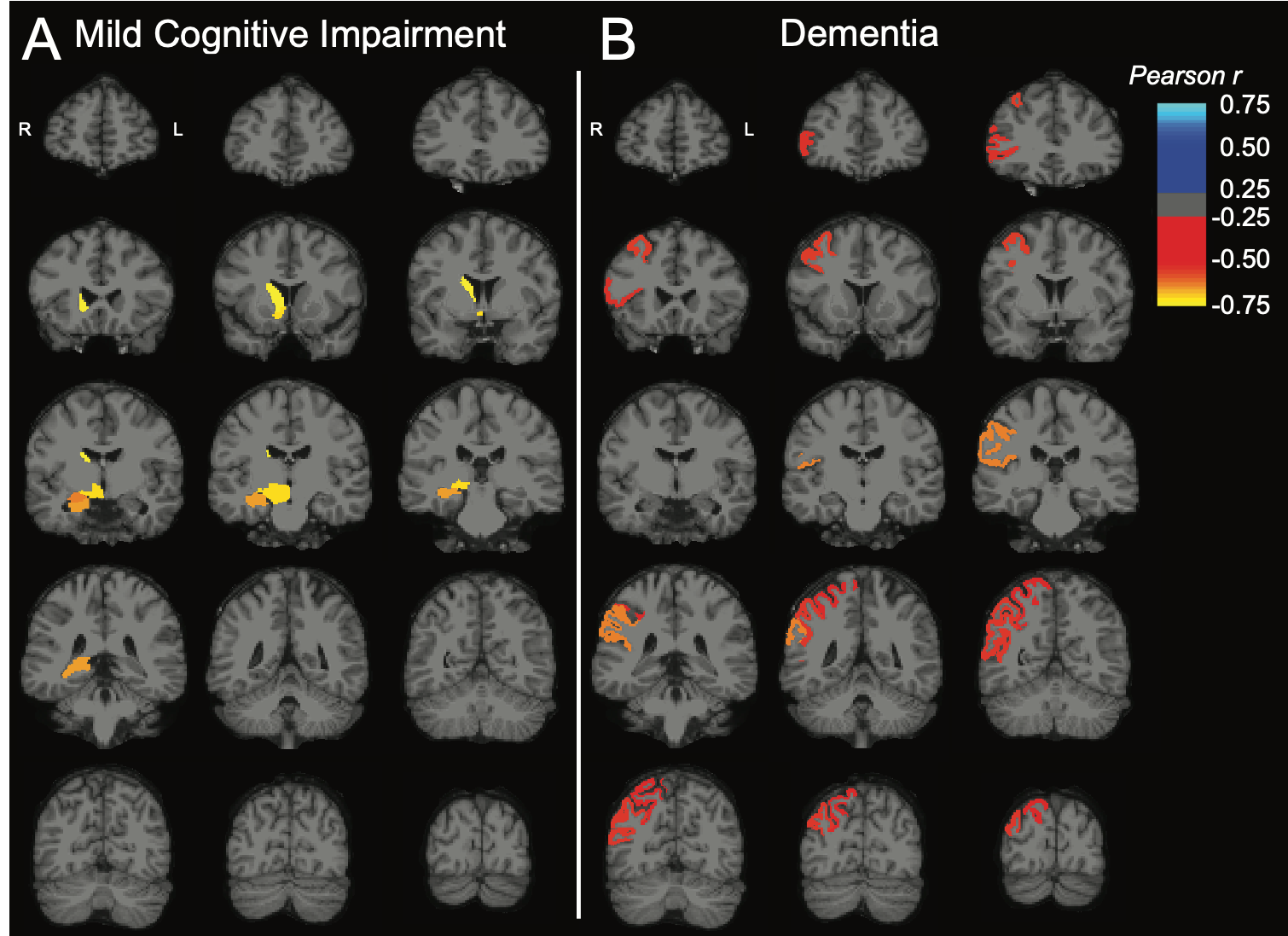


**Supplementary Figure 5. Brain maps of correlations between global *Aβ* deposition and SV2A after IY-PVC.** After application of IY-PVC to [^11^C]UCB-J and [^11^C]PiB *DVR* parametric images, brain maps were created by producing images with the voxels in each FreeSurfer region set uniformly to the calculated Pearson *r* for that region and overlaid on an MNI template T1 MRI. Correlations were across all 84 lateralized FreeSurfer brain regions and displayed only for regions with uncorrected *P* < 0.05 in both the (A) aMCI and (B) dementia diagnostic groups. MR image slices adhere to radiological convention, with orientation denoted in the first coronal section of each image series. Similar to non-PVC data, (A) inverse associations with global Aβ deposition were suggested with SV2A in right-sided subcortical and medial temporal structures in participants with aMCI, (B) as were inverse associations with global Aβ deposition and SV2A in right-sided frontal, temporal, and parietal cortical regions in the dementia group. Abbreviations: *DVR*, distribution volume ratio using a whole cerebellum reference region; Aβ, amyloid beta; aMCI, amnestic mild cognitive impairment.

| **Supplementary Table 5. Correlation of regional *Aβ* deposition and regional SV2A in aMCI and dementia after IY-PVC** | | | | |
| --- | --- | --- | --- | --- |
|  | **Mild Cognitive Impairment (n = 14)** | | **Dementia (n = 24)** | |
| **Primary Region** | **Pearson *r*** | ***P*** | **Pearson *r*** | ***P*** |
| **Hippocampus** | 0.43 | 0.12 | 0.62 | 0.001* |
| **Exploratory Regions** | **Pearson *r*** | ***P*** | **Pearson *r*** | ***P*** |
| **Entorhinal** | -0.03 | 0.92 | 0.32 | 0.13 |
| **Parahippocampal** | -0.42 | 0.14 | 0.20 | 0.36 |
| **Amygdala** | -0.01 | 0.98 | 0.36 | 0.09 |
| **Lateral Temporal** | -0.26 | 0.37 | -0.08 | 0.71 |
| **Prefrontal** | 0.00 | 1.00 | -0.12 | 0.59 |
| **PCC/Precuneus** | -0.07 | 0.81 | -0.20 | 0.36 |
| **Anterior Cingulate** | 0.11 | 0.71 | -0.01 | 0.96 |
| **Lateral Parietal** | -0.05 | 0.86 | -0.16 | 0.45 |
| **Lateral Occipital** | 0.05 | 0.86 | -0.25 | 0.25 |
| **Medial Occipital** | 0.02 | 0.94 | -0.43 | 0.04* |
| **Pericentral** | 0.17 | 0.57 | 0.03 | 0.89 |

Data are Pearson *r* and associated two-tailed *P* values obtained from separate univariate linear regression analyses in each group, uncorrected for multiple comparisons. After application of IY-PVC, regional Aβ burden ([^11^C]PiB *DVR*) was correlated with synaptic density ([^11^C]UCB-J *DVR*) within each of the 4 medial temporal structures and 8 neocortical ROIs included in the primary analysis. The “paradoxical” positive correlation between regional Aβ deposition and SV2A binding in the hippocampus of participants with aMCI was again demonstrated after correction for partial volume effects. [Note: As with the non-PVC data, this correlation was also present if the aMCI and dementia samples were combined: *r* = 0.54, *P* = 0.0004]. In addition, the nominal inverse association between Aβ deposition and SV2A binding in the medial occipital ROI in participants with dementia was again observed (*r* = -0.43, *P* = 0.04). * denotes *P* < 0.05. Abbreviations: IY-PVC, Iterative Yang Partial Volume Correction; PCC, posterior cingulate cortex; Aβ, amyloid beta; PiB, Pittsburgh Compound B; SV2A, synaptic vesicle glycoprotein 2A; *DVR*, distribution volume ratio using a whole cerebellum reference region; ROI; region of interest; aMCI, amnestic mild cognitive impairment.


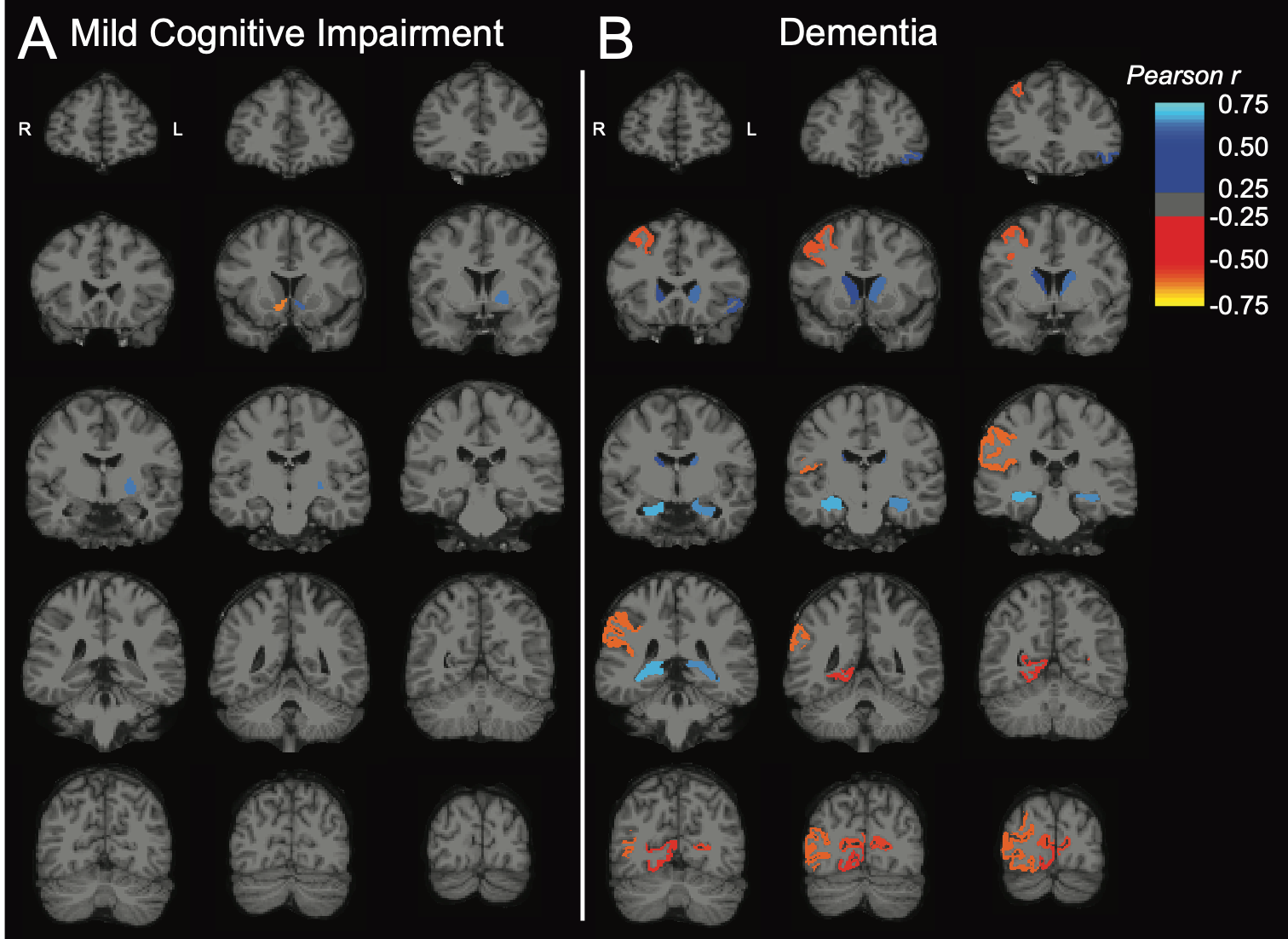


**Supplementary Figure 6. Brain maps of correlations between regional *Aβ* deposition and SV2A after IY-PVC.** After application of IY-PVC to [^11^C]UCB-J and [^11^C]PiB *DVR* parametric images, brain maps were created by producing images with the voxels in each FreeSurfer region set uniformly to the calculated Pearson *r* for that region and overlaid on an MNI template T1 MRI. Correlations were across all 84 lateralized FreeSurfer brain regions and displayed only for regions with uncorrected *P* < 0.05 in the (A) aMCI and (B) dementia diagnostic groups. MR image slices adhere to radiological convention, with orientation denoted in the first coronal section of each image series. Similar to non-PVC data, (A) Few correlations were observed in aMCI participants, (B) while in participants with dementia, inverse associations between regional Aβ deposition and SV2A binding were strongest in the right-hemisphere and included frontal (right caudal middle frontal gyrus), temporal (right supramarginal gyrus), and occipital (right pericalcarine, right lingual, and left pericalcarine) cortical regions. Notably, positive correlations in the bilateral hippocampi of the dementia group were again suggested after correction for partial volume effects. Abbreviations: *DVR*, distribution volume ratio using a whole cerebellum reference region; Aβ, amyloid beta; aMCI, amnestic mild cognitive impairment.
